# A microfluidic platform reveals the role of liquid movement on the dispersal of *Pseudomonas putida* via *Pythium ultimum* hyphal highways

**DOI:** 10.1093/femsml/uqag035

**Published:** 2026-09-21

**Authors:** Eleonora Moratto, Emily Masters-Clark, Amelia J Clark, Aliya Pachmann, Cameron W M Murphy, Saskia Bindschedler, Pilar Junier, Claire E Stanley

**Affiliations:** Department of Bioengineering, Imperial College London, London SW7 2AZ, United Kingdom; Department of Bioengineering, Imperial College London, London SW7 2AZ, United Kingdom; Department of Bioengineering, Imperial College London, London SW7 2AZ, United Kingdom; Department of Bioengineering, Imperial College London, London SW7 2AZ, United Kingdom; Department of Civil and Environmental Engineering, Imperial College London, London SW7 2AZ, United Kingdom; Centre for Environment and Life Sciences, CSIRO Land and Water, Private Bag No 5, Wembley WA 6913, Australia; Laboratory of Microbiology, University of Neuchâtel, Neuchâtel CH-2000, Switzerland; Laboratory of Microbiology, University of Neuchâtel, Neuchâtel CH-2000, Switzerland; Department of Bioengineering, Imperial College London, London SW7 2AZ, United Kingdom

**Keywords:** microfluidics, fungal highway, microscopy, bacterial dispersal, hyphal networks, liquid transport, hyphal highway

## Abstract

Hyphal networks contribute to the connectivity between discrete soil microbial populations by providing a physical network through unsaturated soil matrixes. This network serves as a scaffold for bacteria to disperse and access previously isolated spaces via the ‘fungal highway’. As bacteria require a liquid film for flagellar swimming, liquid saturation is a significant limiting factor for their dispersal in soil. The current working hypothesis is that bacteria can utilize thin liquid films associated with fungal or fungal-like hyphae to cross air gaps in soil environments. Studying these interactions at the microbial scale, however, is challenging due to the complex and stochastic ‘black box’ nature of soil environments. Hence, the microfluidic Hyphal Highways Device was developed to accomplish long-term quantification of bacterial dispersal along hyphal networks at single-cell level. Key findings highlight that *Pythium ultimum* is essential for *Pseudomonas putida* movement across an unsaturated environment, with mycelial biomass and liquid transport being key factors influencing bacterial dispersal. Additionally, *P. putida* actively modified the distribution of the transported liquid, increasing liquid patch connectivity. The Hyphal Highways Device provided quantitative insights into hyphal-mediated bacterial dispersal, opening new opportunities to study microscale interactions that shape microbial dynamics in natural and agricultural soils.

## Teaser

The Hyphal Highways Device provides quantification of bacterial dispersal along hyphal networks at single-cell level, revealing active modification of liquid architecture on *Pythium ultimum* hyphal highways by *Pseudomonas putida* bacteria.

## Introduction

One of the central challenges in microbial ecology is to understand how microbial diversity is structured and maintained, and how this diversity underpins essential ecosystem functions such as nutrient cycling and carbon storage (Singh 2015, Delgado-Baquerizo et al. 2016). Hyphal-mediated microbial dispersal is emerging as a key mechanism influencing ecological processes, including resource access, competition, and gene transfer across diverse environments (Erktan et al. 2020, Dubey et al. 2021). Fungal highways (FHs) is a term used to describe the dispersal of prokaryotes along hyphae belonging to Fungi (Kohlmeier et al. 2005, Jansa and Hodge 2021). However, other fungal-like organisms such as Oomycetes also possess hyphae that function as highways. More recently, the term hyphal highways (HH) has been adopted as a more appropriate descriptor for this phenomenon (Pawlowska 2024). The ability of mycelial networks to occupy high volumes of the environment and connect spatially separated micro-niches means that microorganisms can disperse further than their own motility might otherwise allow and expand the range over which they are functionally active. Mycelial networks are well adapted for growth in heterogeneous substrates, such as soil, where hyphae can bridge liquid unsaturated environments and air-filled pores (Sun B et al. 2020a). This, coupled with hyphae’s ability to transport liquid (Allen 2007, Clark et al. 2024), facilitates the movement of other microorganisms, which are limited in their dispersal by the need for a continuous liquid film (Álvarez-Barragán et al. 2023).

Despite our significant understanding of the ecosystem functions of HHs, we lack insight into the processes that drive these interactions at the cellular level. Current methods for investigating HHs use three types of devices: (i) 3D-printed, milli- and microfluidic devices mimicking specific physicochemical aspects of the ecosystem of interest (Simon et al. 2015, Simon et al. 2017, Junier et al. 2021, Mafla-Endara et al. 2021, Kuhn et al. 2022, Buffi et al. 2023), (ii) agar plate-based methods (Zhang et al. 2018, Xiong et al. 2022), and (iii) microcosms (Warmink et al. 2009, Yang et al. 2017, Jiang et al. 2021). Despite the difference in scale and complexity, they are all based on the presence of two compartments: one inoculated with the mycelium and the dispersing prokaryotes of interest, and one containing the target medium, which are then separated by a physical barrier that cannot be crossed by the dispersing microorganism alone. Sampling of the target medium is then carried out to assess HH occurrence. While all these methods have proven invaluable in identifying which organisms form HHs and what environmental factors influence HHs, few allow HH observation at a cellular level (Mafla-Endara et al. 2021, Buffi et al. 2023, Buffi et al. 2025). This mainly stems from the challenging environment in which HH occur. Although HHs occur in a variety of environments, including the phyllosphere, decaying wood, and cheese, soil is the most characteristic and ecologically significant environment to investigate, given the abundance and diversity of bacteria present. Soil is physically, chemically and biologically complex as well as opaque, all significant barriers to direct observation (Stanley and van der Heijden 2017). In most cases, plate-based assays cannot restrict hyphae to a single optical plane, resulting in overlapping hyphae and limited hyphal resolution. Furthermore, agar-based assays lack optical transparency, focal confinement and present a continuous hydrophilic environment, making them less informative for studies on how HHs impact connectivity between discrete soil microbial populations under unsaturated conditions.

In recent years, microfluidic devices have been used to reproduce key aspects of soil, offering precise control over physical and chemical conditions (Stanley et al. 2016). They are well suited to biological applications, and many have been developed for the specific study of fungi and their interactions (Richter et al. 2022). Microfluidic devices offer an excellent platform for these investigations by enabling real-time, high-resolution imaging at the microscale. In addition, bespoke device geometry allows specific behaviours or interactions to be elicited and environmental parameters to be precisely controlled. Several Lab-on-a-Chip technologies were designed to quantify various aspects of filamentous microbial biology, including hyphal tip force (Sun Y et al. 2020b) or fungal space searching and foraging strategies (Held et al. 2019). Furthermore, interaction chips have been developed for the study of bacterial-fungal interactions (BFIs) (Stanley et al. 2014) as well as fungal–fungal interactions (FFIs) (Gimeno et al. 2021). The BFI device was designed to accommodate inoculation of fungal hyphae and bacteria through separate inlets and observe their interactions in microchannels. This enabled quantification of parameters such as changes in hyphal growth and revealed *Bacillus subtilis* NCIB 3610 attachment to a specific subset of *Coprinopsis cinerea* hyphae, inhibiting hyphal growth and initiating blebbing of hyphal cells (Stanley et al. 2014). However, the BFI device is not suitable for HH observation because it was designed to accommodate full channel saturation with liquid media. Using the FFI device, Gimeno et al. (2021) showed that *Trichoderma rossicum* and *Fusarium graminearum* hyphae can grow across unsaturated channels and would therefore be able to bridge the gaps between liquid-saturated soil micro-niches. Furthermore, Clark et al. (2024) used the FFI device to observe and quantify liquid transport by *Pythium ultimum* hyphae across unsaturated channels, suggesting that hyphae might play an active role in bridging these gaps, given that a 2–10 μm-thick liquid film surrounding hyphae is necessary for bacterial transport (Jiang et al. 2021).

In this paper, we develop the Hyphal Highways Device (HHD), a microfluidic device that allows long-term observation of bacterial dispersal along hyphae in unsaturated conditions. We then used this device to quantitatively describe *Pseudomonas putida* movement along *P. ultimum* hyphae, model organisms often employed for the study of HHs (Furuno et al. 2010, You et al. 2022, Buffi et al. 2025). We show that the presence of *P. ultimum* is necessary for *P. putida* transport across an unsaturated channel and demonstrate that mycelial biomass and hyphal front length are determining factors in the bacteria’s ability to reach the outlet. We also show that the transport of environmental liquid by *P. ultimum* mycelium drives the HHs when interacting with *P. putida* and that the bacteria modify the distribution of the transported liquid. We confirm that liquid is necessary for HHs and show that *P. putida* increases the likelihood of liquid patch fusion.

## Materials and methods

### Microbial culture conditions

Mycelia from the Oomycete *P. ultimum* M194 (Supplementary Table 1) were cultured on potato dextrose agar (PDA) (Thermo Fisher Scientific) at 26°C. When the experiment required it, fluorescein sodium (Sigma–Aldrich) was added to the medium with a final concentration of 6.6 µM (Clark et al. 2024). Fresh active cultures on PDA were prepared 3 days prior to each device inoculation to ensure actively growing mycelium. Active cultures were prepared by cutting plugs from the apical edge of *P. ultimum* mycelia using a cork borer (diameter = 4 mm) from the growing edge and placing them in the centre of fresh PDA, then incubated at 26°C for 3 days. Importantly, cultures were grown on thick agar (25 ml media into each 10 × 10 cm Petri dish) to yield tall agar plugs. This increases the longevity of the experiment by maintaining humidity and nutrients throughout the protocol and also these plugs remain intact to the end of the experiment to be sampled.

*P. putida* strain KT2440::gfp was kindly provided by the Department of Biotechnology and Biomedicine (Technical University of Denmark, Denmark) (Supplementary Table 1). *P. putida* F1 mScarlet-I (pMRE135) (Supplementary Table 1) was generated as described in the supplementary methods. Both strains were maintained on nutrient agar (NA) (Oxoid) plates supplemented with gentamicin (10 µg/ml) or kanamycin (50 µg/ml), respectively. Bacterial overnight liquid cultures were prepared in 5 ml nutrient broth (NB) (Oxoid) supplemented with gentamicin (10 µg/ml) or kanamycin (50 µg/ml), as appropriate. Cultures were inoculated from a single colony and incubated overnight at 25°C with shaking at 200 rpm.

### Device design and fabrication

A digital 2D photomask containing the device design was generated using AutoCAD. Master mould fabrication was performed using standard photolithography techniques. Briefly, a 9 cm silicon wafer was cleaned with solvents and oxygen plasma, then spin-coated (WS-650MZ Modular, Laurell Technologies Corporation) with SU-8 2010 photoresist (Kayaku Advanced Materials) up to 3000 rpm (ramp 350 rpm/s) for 40 s to achieve a height of 10 µm. The coated wafer was soft baked (65°C for 1 min, 95°C for 3 min), before patterning using a maskless system (Microlight3D) (85% power, 128.5 s exposure per frame). Following UV exposure, the wafer was baked once more (65°C for 1 min, 95°C for 4 min) before uncured SU-8 was removed using developer solution for 2 min. The wafer was then silanized for 2 h under a vacuum using 50 μl of chlorotrimethylsilane (Sigma–Aldrich).

Standard soft lithography was used to cast poly(dimethylsiloxane) (PDMS) and manufacture HHDs. Briefly, PDMS (Sylgard 184 elastomer kit, VWR) was mixed in a 10:1 ratio of base to curing agent and degassed for 1 h in a vacuum chamber to remove bubbles. This was then poured onto the centre of the master mould and cured at 70°C overnight. Glass-bottomed Petri dishes (World Precision Instruments; Ø dish = 50 mm, Ø glass = 23 mm) were coated with 0.5 g of PDMS using a NiLO spin coater (500 rpm, 25 ms^−1^ acceleration, for 50 s) and cured at 70°C for 2 h.

The cured PDMS was removed from the mould, cut into individual slabs and the inlets punched using a precision cutter (SLS; diameter = 4.75 mm). The PDMS slabs were then washed: 5 mins sequential sonication in 0.5 mM NaOH, 70% EtOH, and finally sterile Milli-Q water, rinsing with sterile water between each step. The PDMS-coated Petri dishes were also washed, but without the sonication and incubation; these were simply filled with each washing reagent in sequence to minimize any interaction with the plastic outer edge. The PDMS slabs and coated Petri dishes were dried with filtered pressurized air and left for ∼1 h at 70°C. Once dry, they were next placed into a plasma oven and treated with air plasma for 1 min (Diener Electronic, Germany; vacuum pressure 0.75 mbar, power 50%), after which they were removed and immediately bonded together to form the HHD. The fabricated HHD was then placed back into the oven at 70°C for 48 h to ensure hydrophobic recovery of the PDMS. Devices were cooled to room temperature before inoculation.

### Device inoculation

*P. ultimum* inoculant agar plugs were cut from the growing edge of the cultured mycelium using a sterile 4 mm-diameter borer and placed into the inlet, mycelium side down (corresponding to 0 days post inoculation—dpi). An uninoculated PDA plug was placed in the outlet and 100 µl of sterile Milli-Q water was added to the area of the Petri dish surrounding the HHD to maintain a humid environment. When liquid visualization was desired, the PDA plugs were supplemented with fluorescein. The inoculated devices were then incubated at 26°C overnight to allow the mycelium to begin colonizing the channel.

The next day (1 dpi), the desired bacterial culture was centrifuged at 2000 rpm for 10 mins. After centrifugation, the supernatant was discarded, and the pellet resuspended in 5 ml sterile 1X phosphate-buffered saline (PBS) (90% Milli-Q water, 10% Gibco™ PBS, pH 7.2). This washing step was repeated twice to remove the culture media, and the final pellet was resuspended in 3 ml of sterile PBS to concentrate bacteria. The OD600 of this final suspension was read and then adjusted to a final optical density of 1.6. Ten microlitres of this bacterial suspension was pipetted directly onto the top of the pre-cut *P. ultimum* plug inoculated at 0 dpi in the HHD inlet. Then, a fresh and sterile PDA plug was placed on top in contact with the mycelial plug and the device incubated at 26°C for the remainder of the experiment. *P. putida* KT2440::gfp was used for all experiments, unless liquid visualization was desired, in which case *P. putida* F1 mScarlet-I was used.

### Imaging and image analysis

To characterize HH behaviour, devices were imaged every 24 h (1–5 dpi) using an inverted microscope (Eclipse Ti-U and Eclipse Ti-2, Nikon). The entire HHD channel was imaged by tile scanning at 20× using filters (Supplementary Table 2) to image phase contrast (PC), green fluorescence (FITC), and red fluorescence (TRITC). Mycelial, bacterial, and liquid locations were quantified from the tile scans using an ImageJ(Fiji) (Schindelin et al. 2012) macro developed for this project (Supplementary Macro 1 and 4). Briefly, a line matching the HHD channel thickness was drawn along the channel, and manual thresholding was applied to the PC image to distinguish the mycelial structure from the background. The average pixel intensity was measured along the line for PC, FITC, and TRITC. The FITC and TRITC values were then normalized based on background fluorescence. All data was analysed in R Studio, using packages ggplot2, dyplr, and matrixStats. We also acquired exploratory videos or images at 40×, switching between PC, FITC, and TRITC, using in-built autoLUT software.

The hyphal and liquid fronts were measured manually using ImageJ (Fiji) (Schindelin et al. 2012). The hyphal front was defined as the distance from the inlet to the tip of the hyphae that was closest to the outlet. To measure this, the line tool was used to draw a line parallel to the channel from the inlet to the tip of the hyphae. The liquid front was defined as the distance from the inlet to the edge of the closest liquid patch to the outlet. The same measurement method was applied for both fronts.

Another macro (Supplementary Macro 5) was developed in ImageJ(Fiji) (Schindelin et al. 2012) to quantify liquid patch formation and bacterial distribution at the liquid front. This macro quantifies experiments where liquid was stained with fluorescein and the bacteria used was *P. putida* F1 mScarlet-I. Briefly, each liquid patch is identified by converting the FITC channel to mask and running the *analyse particles* command. This identifies each patch as a region of interest (ROI) and returns each area and distance from the inlet. The ROIs are then overlapped on the TRITC channel, and the average pixel intensity is measured for each patch. This returns a proxy of the bacterial biomass in each liquid patch. Given that the macro is unable to recognize when the channel is fully saturated with liquid, this data was manually substituted with an area and average pixel intensity corresponding to the entire channel.

We manually quantified the number of HHs using ImageJ (Fiji) software (Schindelin et al. 2012). An HH was defined as any hypha that is surrounded by a liquid film (as determined through quantifying the fluorescence in the FITC channel), irrespective of the presence of bacteria. To count the HHs in each image, we utilized the cell counter tool.

### Bacterial transport quantification

Colony-forming units (CFUs) counting was used to quantify the number of bacteria that traversed the HHD. A sterile 4-mm-diameter borer was placed into the target inlet and twisted to sever the hyphae growing onto the target plug from the channel. The target plug was removed and placed in 1 ml sterile-filtered 1% hexametaphosphate to separate the bacteria that may have associated with the hyphae. Using a pestle or forceps, the agar plug was ground in the water until homogenous. The suspension was pipetted into 9 ml sterile-filtered 1X PBS (90% MilliQ water, 10% Gibco™ PBS, pH 7.2) and vortexed gently. The suspension was filtered twice through 20 µm nylon meshes (Plastok Associated LTD), and once through 5 µm meshes (Plastok Associated LTD). Afterwards, 100 µl of the filtered suspension was pipetted onto an NB agar plate with the appropriate antibiotic and incubated at 27°C for 3 days. CFUs were counted by taking pictures of the plates and using the cell counter tool in ImageJ software.

## Results

### Design and function of the HHD

To observe and quantify how bacteria disperse along hyphal networks, we developed the HHD. This device enables unidirectional growth of hyphae and co-inoculation with bacteria under unsaturated conditions, facilitating time-resolved monitoring of network topology and their relative positions (Fig. 1a–d). To develop and characterize the HHD, we used the HH model organisms *P. putida* KT2440::gfp and *P. ultimum* M194 (You et al. 2022, Buffi et al. 2025).

**Figure 1:**
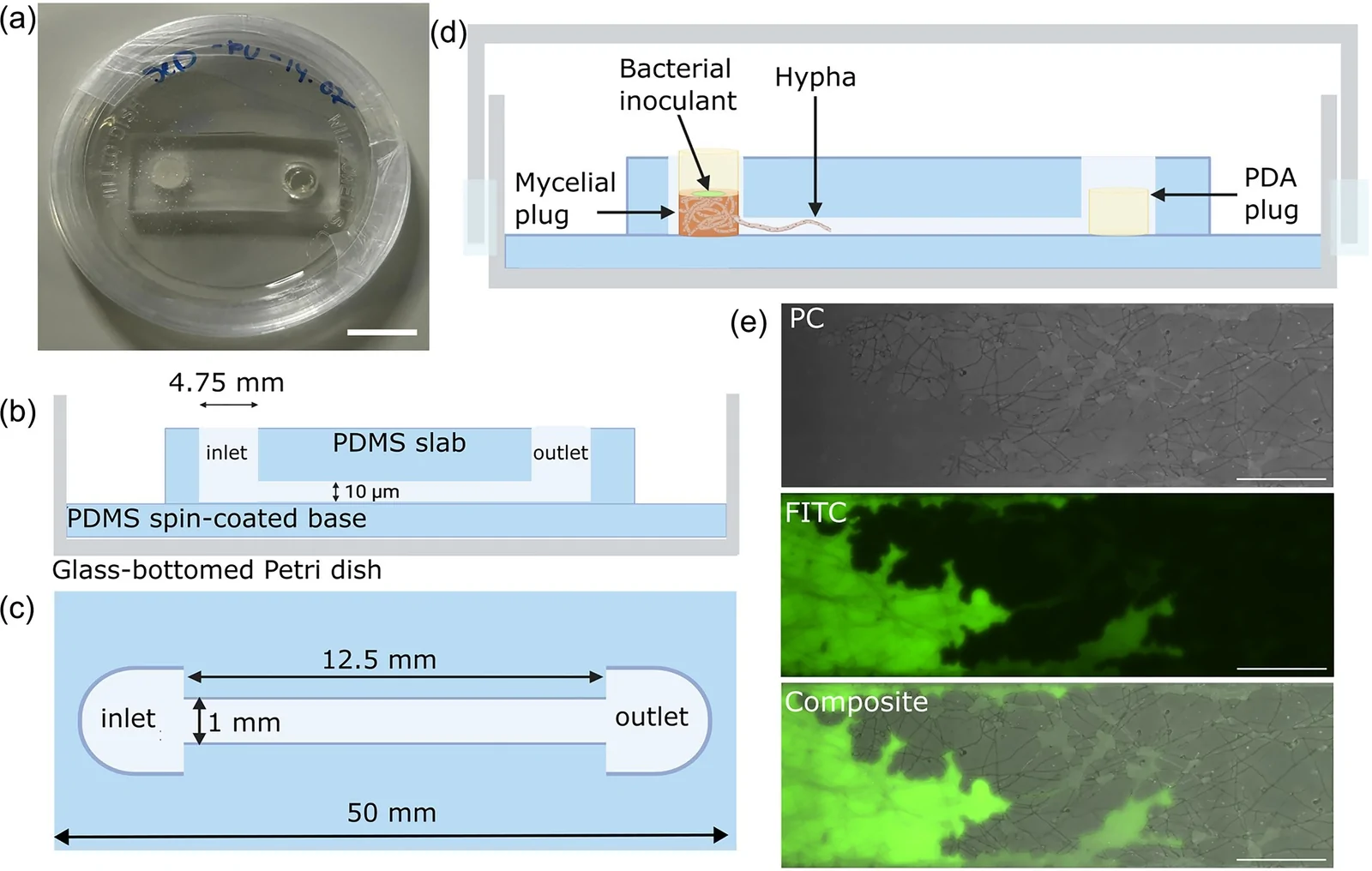
The HHD was designed to visualize bacterial dispersal across hyphal networks under unsaturated conditions. (a) Image of an inoculated HHD showing a poly(dimethylsiloxane) (PDMS) slab with the embossed microchannel bonded to a PDMS-coated glass-bottomed Petri dish. (b) Schematic representation of the HHD, side view (not to scale—dimensions given). The glass-bottomed Petri dish is spin-coated with a thin layer of PDMS, to which the PDMS slab embossed with a 10 µm channel and inlets is bonded. (c) Schematic representation of the HHD, top view (not to scale—dimensions given), which shows the 2D design of the microchannel with dimensions. (d) Schematic representation of the HHD inoculated with both mycelia and bacteria, and a hypha moving through the channel from the inlet to the outlet (containing a fresh PDA plug). The inoculation strategy was designed to allow hyphae to pre-establish in the device for 1 day prior to bacterial co-inoculation. (e) Representative images of a section of the HHD channel inoculated with *Pythium ultimum* and *Pseudomonas putida-*GFP 3 days post-mycelial-inoculation (dpmi). The top panel was acquired using phase contrast (PC) and the middle panel using FITC, enabling the visualization of the mycelium and bacteria, respectively. The bottom panel is the composite image showing the presence of bacteria in liquid patches around hyphae. Scale bars, 500 µm.

The HHD design consists of an observation channel (width: 1000 µm, length: 12 500 µm) to allow for the formation of a substantial mycelial network. We chose to exclude constriction points from channel geometry to prevent disruption of liquid films formed by the hyphae (Fig. 1b and c), as previously reported by Clark et al. (2024). The channel was designed to be 10 µm in height, which has been found to accommodate and confine hyphae on the order of 1–7 μm (Stanley et al. 2014, Gimeno et al. 2021) with *P. ultimum* hyphal diameter ranging between 1.3 and 6.24 μm (Garibaldi et al. 2010). Hyphal or nutrient media plugs can be placed into a 4-mm-diameter inlet or outlet, respectively (located at either end of the main observation channel), allowing for directional growth of the hyphae across the main channel (Fig. 1a–d).

The HHD allows for co-inoculation of mycelium and bacteria in the same inlet by first placing a hyphal plug into the inlet and then inoculating 10 µl of bacterial inoculum on top (Fig. 1d). This is then topped by a second PDA plug, which prevents the bacterial inoculum from drying out as well as providing enough nutrients to sustain growth of both microbes. Importantly, the design allows for the maintenance of an unsaturated, air-filled channel. The unsaturated channel ensures true hyphal-mediated dispersal of bacteria, where liquid is only drawn into the observation channel when hyphae are growing in it. For instance, *P. putida* as a flagellated bacterium would otherwise be motile in the liquid environment provided by a fully saturated channel. To achieve channel unsaturation, the HHD was assembled by bonding the PDMS slab containing the embossed microchannel to a PDMS-coated glass-bottomed Petri dish (Fig. 4d). We hypothesized that the hydrophobic nature of PDMS would prevent full liquid saturation of the microchannel, thus preventing bacteria from entering the channel and reducing condensation. To verify this, we quantified the mean water condensation area observed using both phase contrast (PC) and fluorescence (FITC) imaging. We confirmed that coating of the glass-bottomed Petri dishes with a thin layer of PDMS (0.18 ± 0.10 mm) not only prevented liquid from entering the channel in the absence of hyphae, but also significantly reduced condensation after 48 h (Supplementary Fig. 1a–d). To verify if unsaturated conditions prevented bacterial transport, we performed control experiments by inoculating the device with *P. putida* only and imaging the channel with PC and green fluorescence (FITC) microscopy over 5 days. We observed no presence of bacteria in the channel (Supplementary Fig. 2c). Using the HHD, we were able to visualize the position of fluorescently tagged bacteria relative to their location on the hyphal network (Fig. 1e).

### Developing a quantitative assay to characterize HH

We developed an experimental pipeline to validate the use of HHD in quantifying characteristics of HH, such as mycelial network architecture and density, bacterial dispersal and biomass, hyphal front position, and liquid occupancy of the channel. A *P. ultimum* plug was inoculated in the inlet of a HHD and incubated for 24 h at 26°C to allow the mycelium to begin colonizing the main observation channel. The entire channel was then imaged under PC and FITC filters before inoculating *P. putida* on top of the mycelial plug 1 day later. The device was incubated at 26°C and imaged every 24 h for 5 days. The images obtained show gradual growth of the mycelium across the channel with hyphal front reaching the outlet by day 4 (Fig. 2a). *P. putida* can be observed moving across the channel and reaching the outlet in 5 days when *P. ultimum* is present, but not in the absence of a mycelium (Supplementary Fig. 2b).

**Figure 2:**
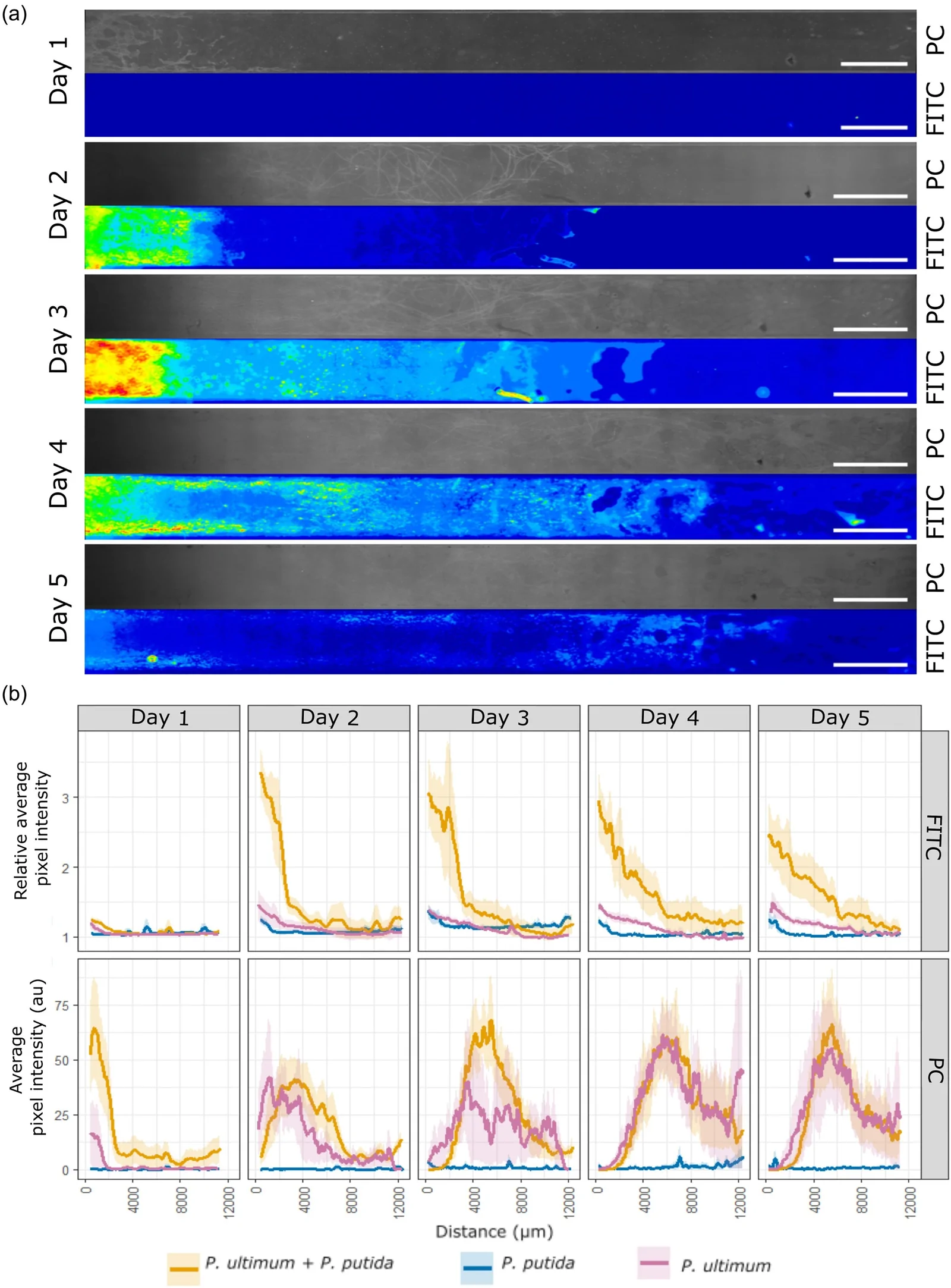
The HHD can be used to quantify bacterial dispersal across hyphal networks. (a) Representative images of the HHD channel inoculated with *Pythium ultimum* and *Pseudomonas putida-*GFP 1–5 days post-mycelial-inoculation (dpmi). Bacteria were inoculated on day 1 after imaging. Top panels are acquired using PC and bottom panels using FITC, enabling the visualization of the mycelium and bacteria, respectively. The FITC image is displayed with a 16-colour LUT look-up table with blue representing low pixel intensity and red representing high pixel intensity. Scale bars, 1 mm. All images depict the inside of the main observation channel only, measuring 12.5 mm in length (Fig. 1c). The images are oriented with the inlet on the left-hand side to match the orientation of the *x*-axis of each graph in panel b. (b) Quantification of the relative pixel intensity of the FITC and PC images (Supplementary Macro 1) along the 12.5 mm microchannel each day of devices inoculated with *P. ultimum* and *P. putida* (orange), *P. putida* only (blue), and *P. ultimum* only (pink). The solid line represents the mean value and the corresponding shaded area represents the standard error (*n* = 8).

To gain further insight into the dynamics of HHs, we developed an ImageJ macro to quantify the average pixel intensity across the channel at each pixel distance from the inlet (Supplementary Macro 1). The information gathered from the FITC and PC channels provides a proxy for biomass of each organism occupying a certain area along the microchannel (Fig. 2b); *P. ultimum* biomass behaved similarly in the presence and absence of *P. putida*, by colonizing the channel over time and reaching the second inlet by day 4. As expected, no green fluorescence was detected on day 1, as the bacteria were inoculated directly after imaging *P. ultimum* on day 1 (Fig. 2b). Green fluorescence was only detected in the channel when both organisms were present, indicating that *P. putida* is unable to traverse unsaturated environments by itself (Fig. 2b). Notably, most of the bacterial biomass was located behind the hyphal biomass, strengthening our hypothesis that hyphae drive bacterial movement (Supplementary Fig. 2c).

A limitation of this method is the slight hyphal autofluorescence observed in the FITC channel; however, *P. ultimum* hyphal autofluorescence (1.155 ± 0.0003 a.u.) is negligible compared to bacterial fluorescence in the presence of *P. ultimum* (1.584 ± 0.001 a.u.) and similar to the background fluorescence detected when the channel is empty (1.049 ± 0.0002 a.u.) (Fig. 2b, FITC Day 4 and 5 red and brown lines). Another limitation is the macro’s inability to detect hyphae when partial saturation occurs. For this reason, the majority of *P. ultimum* biomass appears to be concentrated in the middle of the channel between days 3 and 5. This is only due to liquid from the inlet having saturated the first half of the channel by day 3 and liquid from the outlet being distributed along the mycelium on days 4–5 due to hyphae having reached the outlet (Fig. 2b).

### Characterization of *P. putida* dispersal across the HHD channel via *P. ultimum* hyphae

To ensure the hyphal system could accommodate bacterial dispersal from the inlet to the outlet, we extracted bacteria from the PDA plug and plated them to quantify the CFUs (Supplementary Fig. 1e). We observed *P. putida* colony formation ranging between 3000 and 7000 CFUs only in devices inoculated with both *P. ultimum* and the bacteria (Fig. 3a), further confirming that hyphae are required for bacterial dispersal in unsaturated conditions. However, we only obtained bacterial CFUs from the outlets of 4 out of 8 co-inoculated devices.

**Figure 3:**
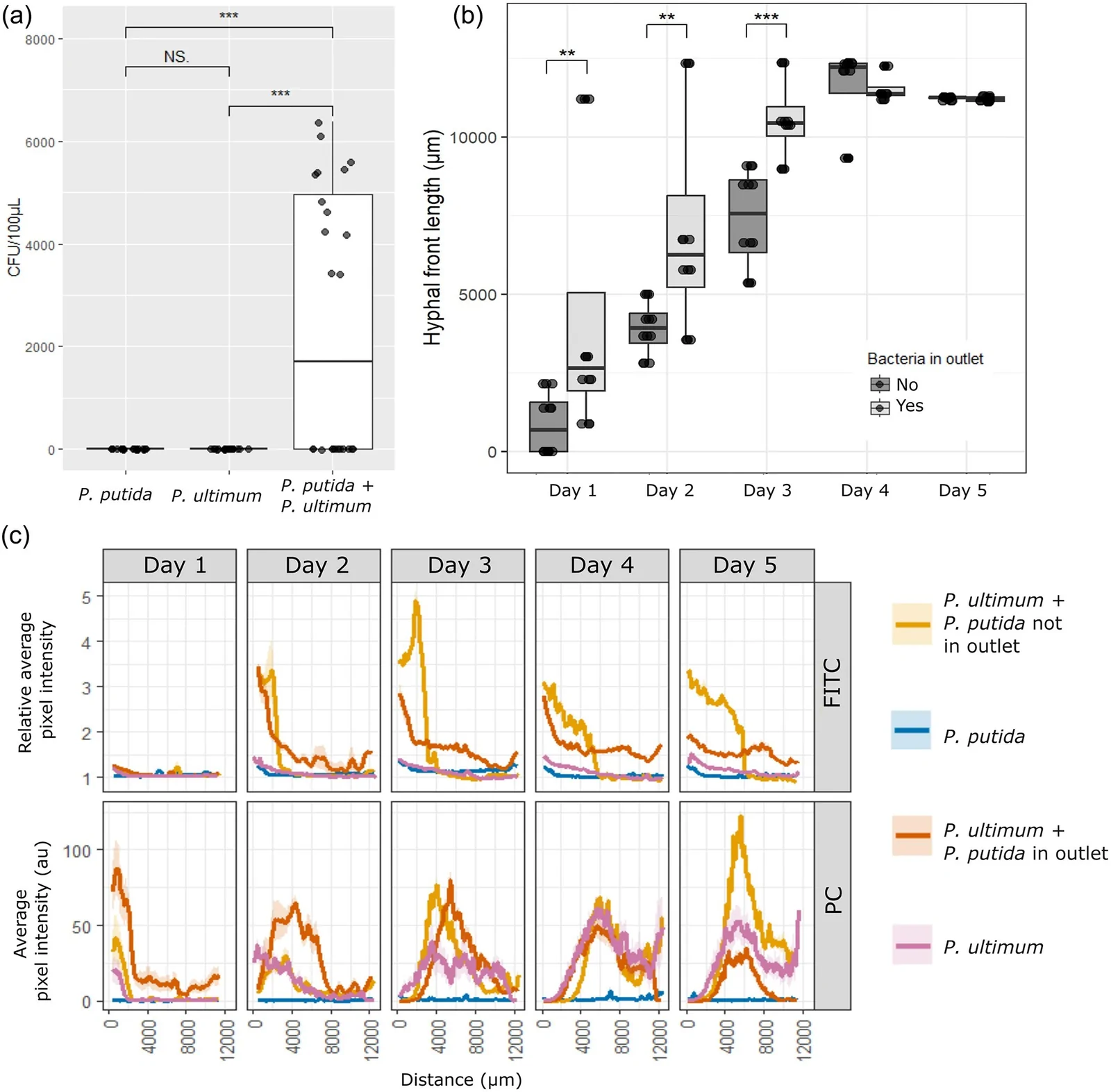
*Pythium ultimum* hyphal networks enable dispersal of *Pseudomonas putida* bacteria across a 12 mm unsaturated space in 5 days. (a) Quantification of *P. putida* colony forming units (CFU/100 µl) extracted from the outlet of HHDs inoculated with (i) *P. putida* only (*n* = 6), (ii) *P. ultimum* only (*n* = 6), and (iii) both *P. ultimum* and *P. putida* (*n* = 8) at 5 dpmi. Three plates were plated per extraction, and each black dot represents the CFU/100 µl of each plate. A Kruskal–Wallis test was used to identify significant differences. Significance is indicated by an asterisk (NS *P* < 0.05; *P  ≤ 0.05; **P  ≤ 0.01; ***P ≤ 0.001). (b) Comparison of hyphal front length from HHDs inoculated with *P. ultimum* and *P. putida* (*n* = 8) split based on the presence (light grey) or absence (dark grey) of bacteria in the outlet. A Dunn’s post-hoc test was run to identify pairwise differences and significance is indicated by an asterisk (*P ≤ 0.05; **P ≤ 0.01; ***P ≤ 0.001). (c) Quantification of the relative pixel intensity of the FITC and PC images (Supplementary Macro 1) along the 12.5 mm microchannel each day of devices inoculated with (i) *P. ultimum* and *P. putida*, where bacteria were present in the outlet (red, *n* = 4); (ii) *P. ultimum* and *P. putida*, where bacteria were absent from the outlet (orange, *n* = 4); (iii) *P. putida* only (blue, *n* = 6); and (iv) *P. ultimum* only (pink, *n* = 6). The solid line represents the mean value and the corresponding shaded area represents the standard error.

To provide further insight into the interaction between *P. ultimum* and *P. putida*, we split our analysis based on the presence or absence of bacteria in the outlet. We observed that when bacteria were detected in the outlet, the hyphal front had progressed significantly further along the channel during the first 3 days of growth (Fig. 3b and c). Hyphae reached the end of the channel by day 4, even when bacteria were not detected in the outlet, indicating that hyphal front positioning alone was not a requirement for bacteria to be detected in the outlet (Fig. 3b and c). We note no difference in the average green pixel intensity of the channel in either case (i.e. regardless of whether or not bacteria were detected in the outlet), indicating that bacterial biomass does not explain the difference in outcome (Supplementary Fig. 3c). The presence of *P. putida* in the channel had no impact on the *P. ultimum* hyphal front or biomass (Supplementary Fig. 3a and b). The difference in hyphal biomass on day 1 can only be attributed to biological variation, since *P. putida* was only added after day 1 imaging. Furthermore, the average PC pixel intensity across the channel, a proxy for hyphal surface area, was higher on days 1 and 2 when *P. putida* reached the outlet (Supplementary Fig. 3c). We also note that when bacteria were not detected in the outlet, the bacterial front did not proceed further than halfway through the channel (Fig. 3c). When comparing the position of the bacterial front fluorescence peak to the hyphal PC peak on day 5, we noticed a higher PC peak in the middle of the device when the bacteria did not reach the outlet (Fig. 3c, Supplementary Fig. 4c). This is supported by the lower *P. ultimum* biomass measured on day 5 (Supplementary Fig. 3c). Liquid saturation in the channel decreases image contrast in the PC channel, rendering the macro less able to detect hyphae (Supplementary Fig. 4a and b). The peak in hyphal biomass detected at the centre of the device on day 5, when bacteria are not found in the outlet (Fig. 3c, Supplementary Fig. 4c), was not due to an increase in mycelial biomass but a result of unsaturated conditions, making the hyphae in the region better detectable by our macro. This suggests that when the outlet contains bacteria, the middle of the channel is completely saturated, thus ‘hiding’ some of the hyphal biomass (Supplementary Fig. 4a–c). Unsaturated conditions remaining in the centre of the device might constitute enough of a barrier to bacterial transport to prevent it from reaching the outlet. This led us to hypothesize that the distribution of liquid films plays a key role in *P. putida* movement along *P. ultimum* hyphae.

### *Pseudomonas putida* uses HHs to travel between discrete liquid patches

To further characterize *P. putida* behaviours moving along *P. ultimum* hyphae and within liquid films, we obtained exploratory real-time videos and images of the hyphal and bacterial front at higher magnification.

We observed that bacteria were never located in air-filled gaps, only within liquid films with a higher concentration at their perimeter (Fig. 4a and b, Supplementary Videos 1–5). When discrete liquid patches were present, bacteria were present mostly just in contiguous liquid patches and absent from discontiguous ones (Fig. 4a, b, e, and f, Supplementary Videos 1, 2, and 5). While we occasionally observed bacteria in discontiguous liquid patches, their frequency was lower (Table 1, Fig. 4f). We frequently visualized active bacterial movement between liquid patches along hyphae (Fig. 4a–c, Supplementary Videos 1–3) as well as pulsing liquid films causing rapid movement of bacteria and occasionally inversion of the bacterial flow (Supplementary Video 5) (Table 1). Cytoplasmic streaming was also present in *P. ultimum* hyphae. Finally, we observed oomycete structures that divided regions with varying bacterial concentrations within otherwise continuous liquid areas (see Fig. 4d and Supplementary Video 4).

**Figure 4:**
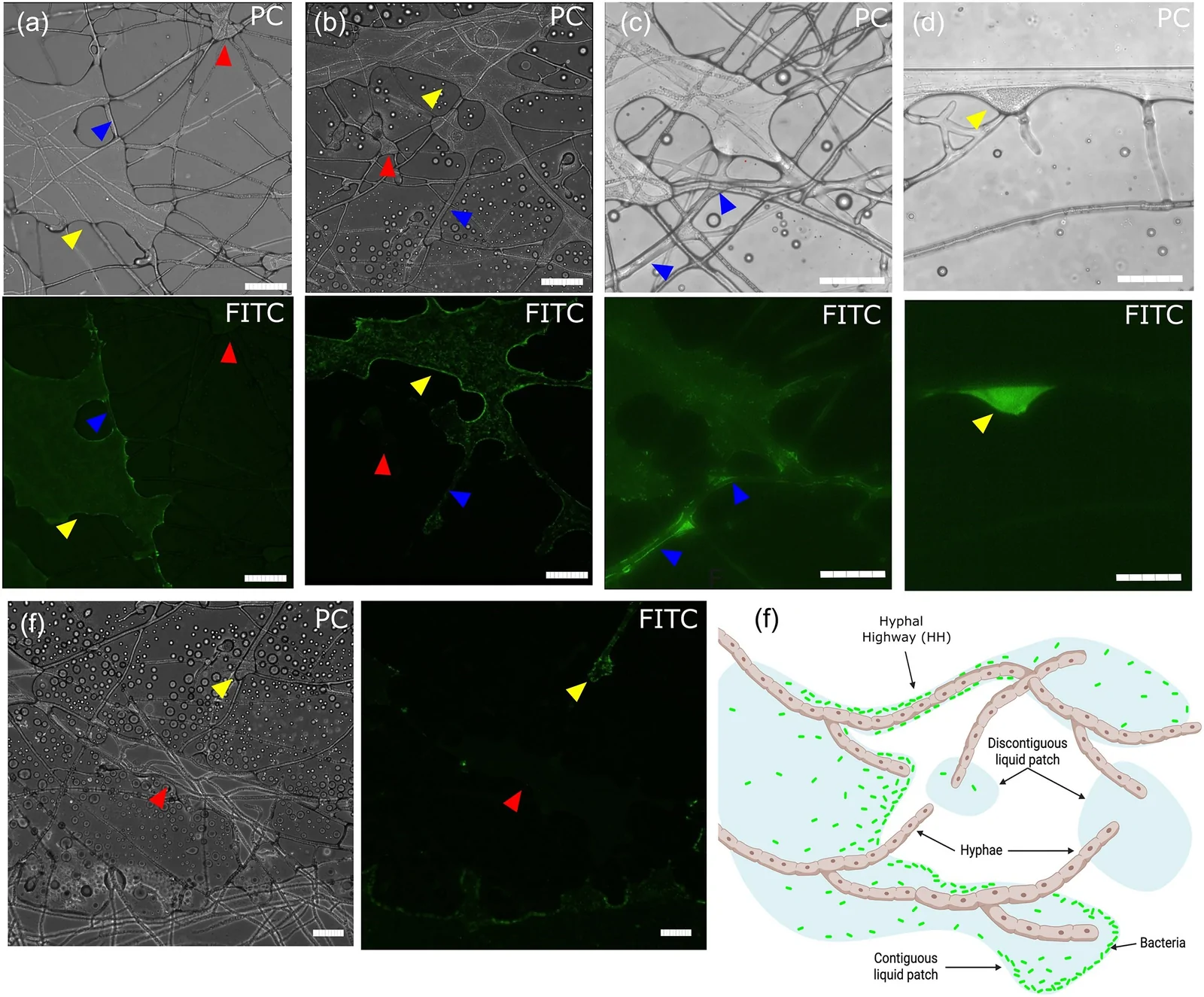
Representative images of HH behaviours. (a), (b), and (c) *Pseudomonas putida* bacteria occupy contiguous liquid film patches along hyphae (yellow arrows) and tend to accumulate at the boundaries of the liquid film. Bacteria are absent from most discontiguous liquid films (red arrow). Bacteria move along narrow liquid films along hyphae (blue arrows). Scale bar, 50 µm. (d) *P. putida* accumulates in liquid films delimited *by Pythium ultimum* structures (yellow arrows). Scale bar, 50 µm. In all cases, the top panel was acquired under PC and the bottom panel with the FITC filter. (e) Bacteria are absent from most discontiguous liquid films (red arrow). The left panel was acquired in PC, and the right panel was acquired with the FITC filter. Scale bar, 100 µm. (f) Schematic representation of the observed behaviours. Bacteria are present in contiguous liquid patches and mostly accumulate at the edge of the liquid. Bacteria invade contiguous liquid patches when connected by hyphae surrounded by a liquid film. In most cases, discontiguous liquid patches are devoid of bacteria. "Hyphal Highway Behvaviours" by A. Pachmann created in BioRender (https://BioRender.com/j4h01nk) is licensed under CC BY 4.0 (https://creativecommons.org/licenses/by/4.0/).

**Table 1:** Summary of observed HH behaviours and their frequency based on a total of 26 observations.

Observed behaviour	Number of observations/26
Bacterial presence exclusively inside liquid films	26/26
Bacterial presence in contiguous liquid patches	26/26
Bacterial presence in discontiguous liquid patches	8/26
Bacteria move between liquid pools along hyphae	20/26
Pulsing of the liquid film	5/26
Cytoplasmic streaming in hyphae	9/26
Bacteria trapped in liquid pools by hyphal structures	8/26

Schematic definition of the behaviours can be found in Fig. 4f.

### *Pseudomonas putida* does not affect *P. ultimum* overall liquid transport

To confirm our observations and better understand the role of liquid movement on the HH, we repeated our experiments using fluorescein to visualize liquid distribution (Clark et al. 2024). Since fluorescein has similar excitation and emission wavelengths to GFP, we used *P. putida* F1-mScarlet to quantify bacterial location (Fig. 5 and Supplementary Fig. 5).

**Figure 5:**
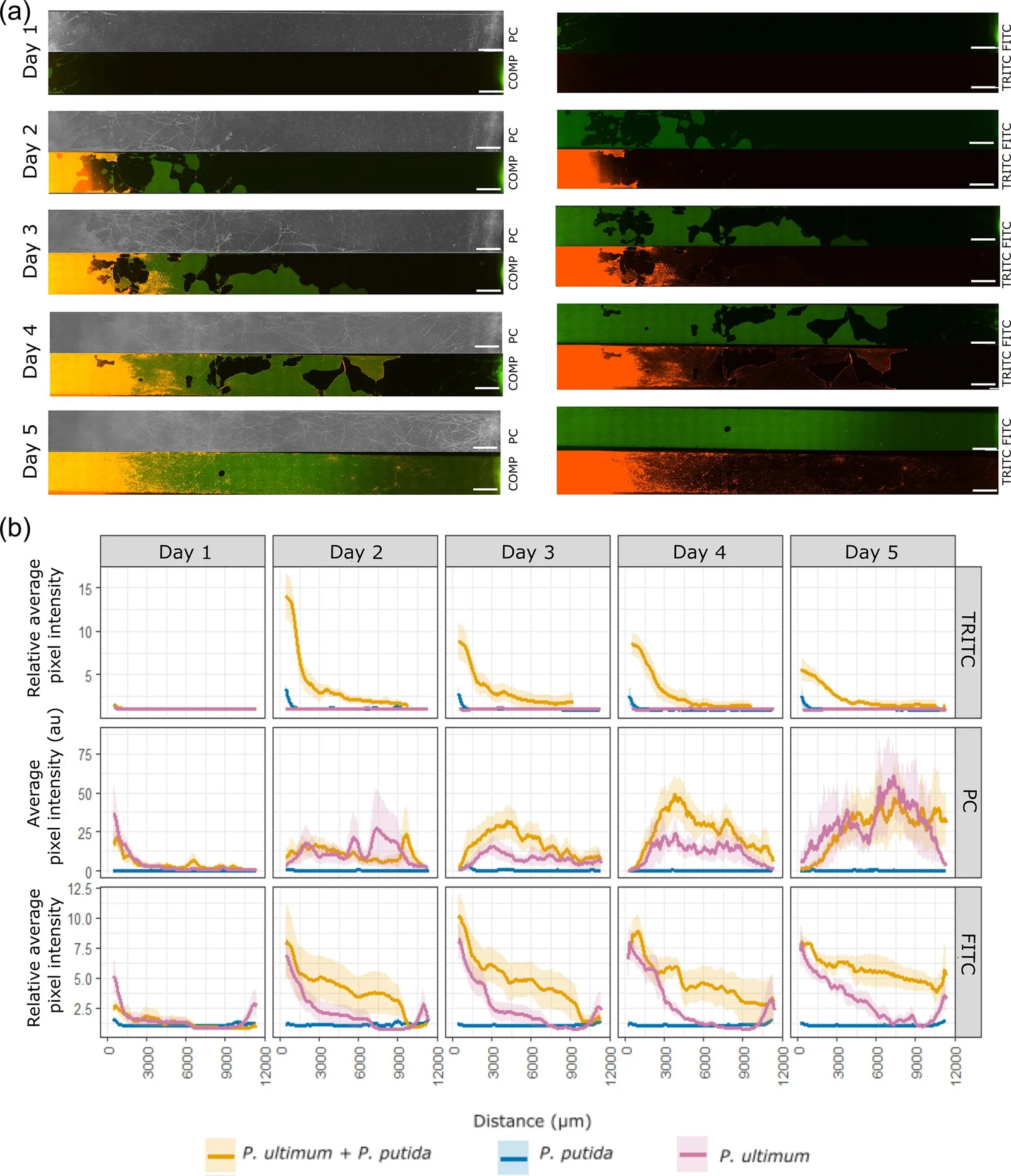
*Pythium ultimum* hyphal networks enable liquid movement and dispersal of *Pseudomonas putida* bacteria across a 12 mm unsaturated space in 5 days. (a) Representative images of the HHD channel inoculated with *P. ultimum* and *P. putida* F1-mScarlet 1–5 days post-mycelial-inoculation (dpmi). Bacteria were inoculated on day 1 after imaging. The left top panels are acquired using PC, the right top panels using FITC, the right bottom panels using TRITC and the left bottom panels are a composite of TRITC and FITC (COMP). PC allows the visualization of the mycelium, FITC of the liquid, and TRITC of the bacteria. The composite image shows the bacteria are only present within liquid films. Scale bars, 500 µm. All images depict the inside of the main observation channel only, measuring 12.5 mm in length (Fig. 1c). The images are oriented with the inlet on the left-hand side to match the orientation of the *x*-axis of each graph in panel b. (b) Quantification of the relative pixel intensity of the TRITC, PC, and FITC images (Supplementary Macro 4) along the 12.5 mm microchannel each day of devices inoculated with *P. ultimum* and *P. putida* (orange), *P. putida* only (blue), and *P. ultimum* only (pink). The solid line represents the mean value and the corresponding shaded area, the standard error (*n* = 6).

We only observed bacterial presence in the channel when *P. ultimum* was also present, and most importantly, *P. putida* could only be found within liquid patches filled areas of the channel (Figs 5 and 6a, Supplementary Figs 5 and 6), confirming what we observed in our exploratory data (Fig. 4 and Table 1). We note that the peak in hyphal biomass is often preceded by high pixel intensity values for liquid position (Supplementary Fig. 5c). This confirms what was previously suggested: the macro is less efficient at detecting hyphae when the channel is saturated with liquid. This further supports our previous findings that unsaturated conditions in the centre of the device might explain bacterial inability to reach the outlet (Fig. 3c, Supplementary Fig. 4c).

**Figure 6:**
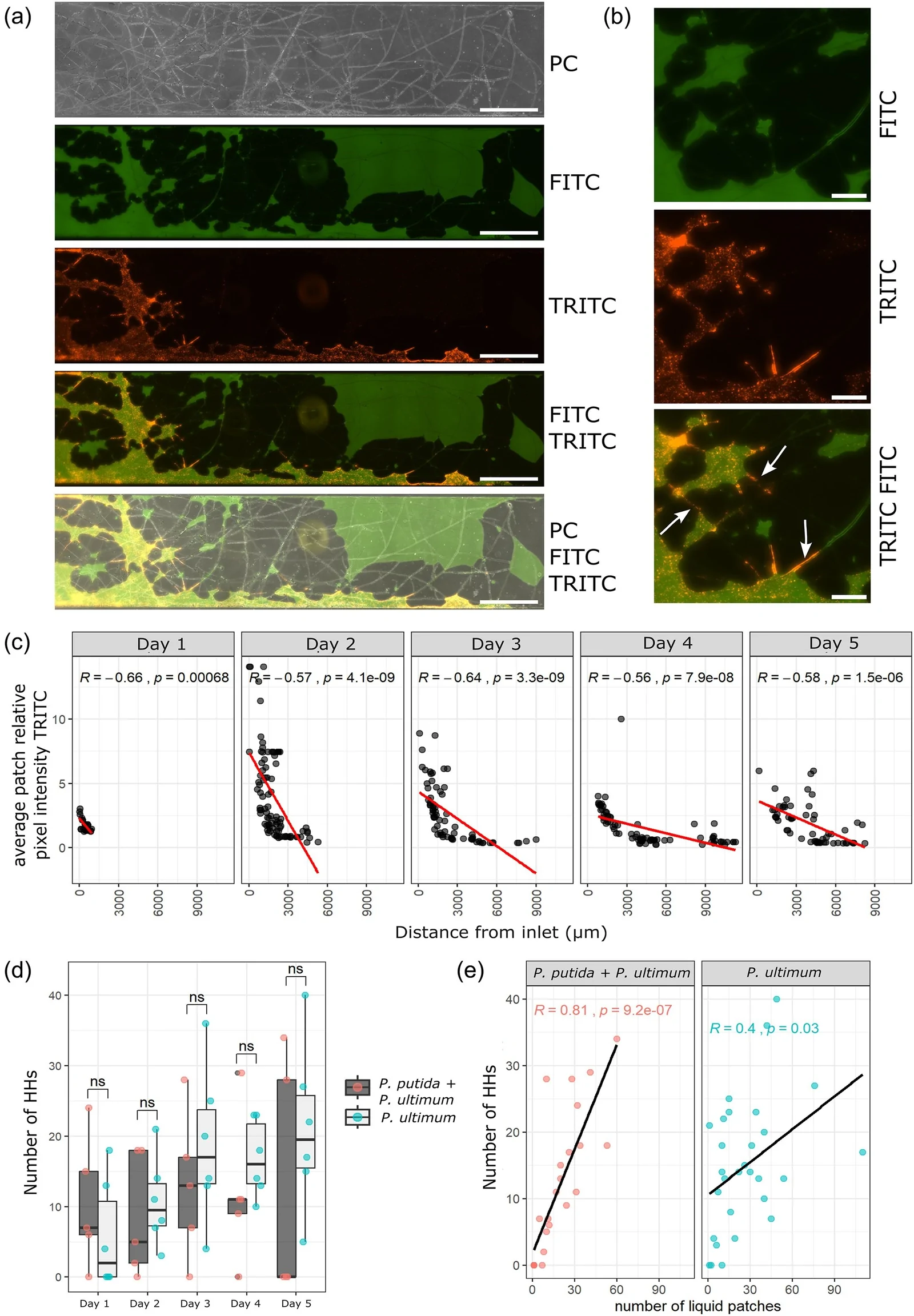
*Pseudomonas putida* concentrates in liquid patches closest to the saturation front. (a) Representative images of liquid patches in the area between the saturation front and the liquid front. The top panel was acquired using PC for the visualization of the *Pythium ultimum* mycelium, the second panel using FITC filter for the visualization of the fluorescein-stained liquid and the third panel using the TRITC filter for the visualization of *P. putida* F1-mScarlet. The fourth panel shows a composite image of the FITC and TRITC images, and the fifth panel is a merge of all images. Scale bar, 500 µm. (b) Zoomed in view of liquid patches showing bacteria located in liquid films surrounding hyphae (white arrows). The top panel was acquired using the FITC filter, the middle panel using the TRITC filter, and the bottom panel is a composite image. Scale bar, 100 µm. (c) Linear regression analysis of normalized TRITC pixel intensity per liquid patch against the distance of the patch from the inlet. Each dot represents one liquid patch. Overall, we find that the position of the liquid patch being closer to the outlet is correlated with a decreased amount of bacteria. Over time, this correlation flattens. (d) Number of hyphae surrounded by narrow liquid films (HHs) connecting liquid patches. Each dot represents the number of HHs in each repeat on one day (*n* = 6); blue dots are data collected from devices with *P. ultimum* only, and red dots from devices with *P. ultimum* and *P. putida* (no significant differences were found). (e) Number of HHs correlates with the number of liquid patches. Each datapoint represents the number of HHs and liquid patches at one timepoint in one repeat (*n* = 6). The left panel shows data collected from devices with *P. ultimum* and *P. putida* with a linear regression line of *R* = 0.81 (*P*-value = 9.2 × 10^−7^). The right panel shows data collected from devices with *P. ultimum* only, with a linear regression of *R* = 0.4 (*P*-value = 0.03). The two regressions are significantly different (Explicit Analysis of Covariance (ANCOVA) test comparing regressions *P*-value: 3.516 × 10^−6^).

We confirmed that, similarly to *P. putida* KT2440::gfp*, P. putida* F1-mScarlet did not affect hyphal biomass or hyphal front (Supplementary Fig. 6a and b). However, it is important to note that the F1-mscarlet-I tag is brighter compared to the GFP tag when expressed in *P. putida* (McCullock et al. 2020), which explains why the TRITC relative pixel intensity was higher for *P. putida* F1-mScarlet compared to the FITC relative pixel intensity for *P. putida* KT2440::gfp. Interestingly, while full saturation of the liquid channel is achieved more often in the presence of bacteria (Fig. 5 and Supplementary Fig. 5), no difference was detected in the average amount of liquid transported by *P. ultimum* (Supplementary Fig. 6a) or the position of the liquid front (Supplementary Fig. 6b). We then wondered if *P. putida* affected the area and distribution of the liquid, in other words, the size and distribution of liquid patches.

### *Pseudomonas putida* impacts liquid distribution

Since bacteria cannot cross air-filled gaps, there is a requirement for liquid patches to be connected to advance towards the outlet. We hypothesized that the presence of bacteria would be associated with fewer, larger liquid patches. To verify this, we wrote and applied a custom macro (Supplementary Macro 5), which identified individual liquid patches in the FITC channel and measured their area and position relative to the inlet. We detected a significant decrease in the number of liquid patches on day five when bacteria were present (Supplementary Fig. 7a). This corresponds to the higher rate of full liquid saturation of the channel we observed when bacteria were present (Fig. 5 and Supplementary Fig. 5). However, when analysing liquid patch areas, we noted that most liquid patches were below 1.0 × 10^5^ µm^2^ in area, while few liquid patches were larger than 1.0 × 10^5^ µm^2^, with the largest being 1.2 × 10^7^ µm^2^ (Supplementary Fig. 7b). These larger patches made contact with one or both side edges of the channel and often corresponded to partial channel saturation. To make sure these effects were due to the presence of bacteria itself and not the addition of 10 µl of liquid where the bacteria are suspended, we ran a control experiment by adding 10 µl of PBS to our experiment without the presence of bacteria. We found no difference in *P. ultimum* and liquid occupancy of the channel (Supplementary Fig. 8a) or in total hyphal biomass and liquid volume (Supplementary Fig. 8b). We did detect a significant difference in liquid front on day 3; however, the same significant difference was present for both the liquid and hyphal front on day 1 before any PBS was added (Supplementary Fig. 8c). This difference can therefore be explained by biological variation between the samples. Furthermore, liquid occupancy and saturation of the channel with the addition of 10 µl PBS are similar to those in the absence of bacteria (Fig. 5b and Supplementary Fig. 8a).

To better understand the effect *P. putida* might have on *P. ultimum* liquid movement, we conducted separate analyses for small liquid patches (≤1.0 × 10^5^ µm^2^) and large liquid patches (>1.0 × 10^5^ µm^2^). This data showed that the presence of bacteria was associated with a size increase of large liquid patches on day 5 (Supplementary Fig. 7c), which numerically describes the full saturation of the channel with liquid (Fig. 5a). The area of smaller patches was unaffected by *P. putida* (Supplementary Fig. 7c). When bacteria were present, both small and large liquid patches were closer to the outlet on day 2, while small liquid patches were further away from the outlet on days 3 and 5 (Supplementary Fig. 7d). A possible interpretation of this data is that the presence of *P. putida* promotes the advancement of both small and large liquid patches in the channel, as well as the fusion of small liquid patches into larger patches, which causes faster channel saturation.

### *Pseudomonas putida* increases HHs’ liquid patch connectivity

Given these findings, we set out to identify a possible mechanism that would promote liquid patch fusion. Similarly to what noted previously, we did not identify bacteria in discontinuous liquid patches (Fig. 6a). We found no correlation between liquid patch area and average amount of bacteria contained in the patch (Supplementary Fig. 7e), but we did identify an inverse correlation between average bacterial amount and distance of the patch from the inlet (Fig. 6c). We also confirmed what has been previously described by Jiang et al. (2021), that bacterial movement along hyphae, i.e. HHs, requires the presence of thin liquid films surrounding the hyphae that connect separate liquid patches (Fig. 6b). We hypothesized that the presence of bacteria might increase the number of HHs connecting liquid patches, thus increasing the chance of patch fusion. To test this, we manually quantified the number of HHs, which remains unaffected by bacterial presence (Fig. 6d). However, the correlation between the number of HHs and liquid patches was significantly different in the presence and absence of bacteria (Fig. 6e, explicit ANCOVA test comparing regressions *P*-value: 3.516 × 10^6^). This implies that when bacteria are present, a more fragmented liquid topology is associated with a higher number of HHs connecting the patches. This indicates that the presence of bacteria increases liquid patch connectivity. We also found that bacteria used 28.79% ± 5.68 of the available HHs and moved bidirectionally across them (Supplementary Video 6).

## Discussion

Here, we present a microfluidic platform to quantitatively investigate HH biology at a cellular level. Compared to previously used methods (Warmink et al. 2009, Simon et al. 2015, Simon et al. 2017, Yang et al. 2017, Zhang et al. 2018, Jiang et al. 2021, Junier et al. 2021, Kuhn et al. 2022, Xiong et al. 2022), the HHD allows direct observation of the mycelial–bacterial interaction in an unsaturated environment, enabling a variety of resolutions from the organism to the cellular level depending on the magnification used. Furthermore, the HHD can also be used to marry quantitative data obtained through image analysis with information derived from sampling the outlet. In particular, the use of a PDMS-coated base renders the entire channel universally hydrophobic. This is essential for investigating true HH behaviour by ensuring hyphal-mediated dispersal of bacteria, particularly when the bacteria of interest are flagellated and can navigate a saturated environment independently. This hydrophobic and unsaturated nature of the HHD provides two important advantages: (i) the reduction in condensation increases image quality, making the HHD ideal for imaging at high magnification, and (ii) hyphal-mediated liquid transport generates more discrete patches akin to a soil environment. In contrast with other microfluidic devices designed to investigate microorganism interactions (Stanley et al. 2014, Held et al. 2019, Sun B et al. 2020a, Gimeno et al. 2021), the wide channel design of the HHD and absence of constriction points allow ample space for mycelial growth, liquid and bacterial movement. Similarly to previous device designs (Stanley et al. 2014, Gimeno et al. 2021, Clark et al. 2024), the HHDs accommodate mycelial plugs as inocula. To ensure consistency and reproducibility, we tightly controlled the amount of media (25 ml) and age of the plates (3 dpi) from which the inocula were taken, as well as always choosing plugs from the growing edge of the mycelium. While better consistency could be obtained using spore inoculation, and this is achievable *on chip* (Richter et al. 2024), it would not be compatible with the unsaturated conditions required for the HHD. This is because spore inoculation requires pumping of liquid into the device (Richter et al. 2024). The presence of an open outlet in the HHD for the sampling of the target media is crucial for providing both confirmation of bacterial transport, as we presented in this paper, but, analogously to columns and millifluidic methods (Simon et al. 2015, Simon et al. 2017, Junier et al. 2021, Kuhn et al. 2022), for the analysis of other parameters such as chemical changes in the environment or gene expression changes in the microorganisms studied.

By coupling the HHD with image analysis, we obtained a precise description of the *P. ultimum–P. putida* interaction dynamics. We confirmed that *P. ultimum* is required for transport of *P. putida* across unsaturated environments (Kohlmeier et al. 2005, You et al. 2022, Buffi et al. 2025) and only identified bacteria within liquid patches. Our observations using fluorescein to visualize liquid confirmed what was previously found by Furuno et al. (2010) that *P. putida* moves within liquid patches and in liquid films around *P. ultimum* hyphae. We also showed that the presence of air gaps interrupting the liquid film in the centre of the channel was a sufficient barrier for *P. putida*, which accumulated at the edge of the air gaps and did not reach the outlet. When liquid patches were not contiguous or connected by hyphae surrounded by thin liquid films, we did not detect bacteria in said patches. Furthermore, discontiguous patches connected by very few hyphae had a lower bacterial concentration.

*P. putida* is a flagellated bacterium and has been shown to mobilize short distances along the water film surrounding hydrophobic textiles such as polyester (Sherry et al. 2023). This might point towards a passive interaction (Kohlmeier et al. 2005), where *P. putida* will occupy any liquid space available, regardless of the presence of *P. ultimum*. This could be confirmed by the absence of an effect of *P. putida* on *P. ultimum* growth. If the interaction were an active one, we could expect the presence of *P. putida* to promote mycelial growth to guarantee movement of more liquid to further locations. For example, *Bacillus subtilis* promotes the growth of its fungal partner *Aspergillus nidulans* by supplying thiamine to the growing hyphae (Abeysinghe et al. 2020). However, we detected no difference in hyphal biomass in the presence of bacteria. Furthermore, the higher hyphal biomass that allows *P. putida* to reach the outlet occurs only 50% of the time, suggesting this is a random phenomenon. However, our observation that *P. putida* alters liquid patch area and connectivity by increasing full-channel saturation suggests some form of active interaction might be occurring. The presence of a higher number of liquid films surrounding hyphae, i.e. HHs, with a greater number of liquid patches, suggests that *P. putida* might increase *P. ultimum* hyphal wettability. *P. putida* bidirectional movement along HHs might be sufficient to increase the likelihood of patch fusion, potentially by expanding the diameter of the HH. However, Buffi et al. (2023) observed a reduction of liquid film thickness around *P. ultimum* in coculture with *P. putida* KT2440, making this an unlikely mechanism. Furthermore, our data does not exclude the presence of a biological interaction. *Pseudomonas spp*. have a documented history of use as biocontrol agents against diseases caused by *P. ultimum* in plants such as soybean. For example, *Pseudomonas fluorescens* has been isolated from soil samples, which showed a decrease in sorghum root rot caused by *P. ultimum* (Idris et al. 2008). Eight *P. putida* isolates, including *P. putida* NIR, have demonstrated the ability to inhibit the growth of *P. ultimum* N1 (Paulitz 1991, Stephens et al. 1993). However, this inhibition only occurs in the presence of seed volatiles from pea and soybean, which typically promote the growth of *P. ultimum* (Pauliz 1991, Paulitz et al. 1992). Stephens et al. (1993) showed that this inhibition is likely due to resource competition where *P. putida* NIR metabolizes these compounds before detection by *P. ultimum* N1, as in the absence of seed volatiles from the media, no inhibitory effect could be observed. Despite the lack of documentation of this phenomenon between these two species, chemical signalling might be present between the two organisms that alters secretion of compounds by the hyphal network and modifies liquid movement, such as hydrophobins and other biosurfactants (Allen 2007, Wosten H and Scholtmeijer 2015, da Silva et al. 2021). While *P. ultimum* (Clark et al. 2024) and other fungi, such as *Agaricus bisporus* (Guhr et al. 2015), *Schizophyllum commune* (Guhr et al. 2016), and *Rhizophagus intraradices* (Kakouridis et al. 2022), have been shown to redistribute liquid, the mechanism remains unclear. Different mycelial organisms possess different properties in their hyphal extracellular matrix that render their surface hydrophilic or hydrophobic and are capable of modifying them through the production of hydrophobins and other biosurfactants (Allen 2007, Wosten H and Scholtmeijer 2015, da Silva et al. 2021). Furthermore, bacteria themselves can produce biosurfactants, which could increase environmental wettability, including hyphae (Eras-Muñoz et al. 2022). This could explain the increase in liquid patch connectivity we observed. While this is likely not the only factor determining liquid movement (Clark et al. 2024), bacterial species have shown preferences for mycelia with different surface properties. For example, various isolates of *Pseudomonas* spp. crossed a hydrophilic surface, using *Alternaria destruens*, but crossed a hydrophobic surface, using *Fusarium pseudonygamai* (Álvarez-Barragán et al. 2023). *P. putida*, however, has been found to travel along hydrophilic mycelia such as *P. ultimum* (Wick et al. 2007). It is not unreasonable to suggest that specific bacterial species could engage in chemical signalling with mycelia to alter their surface chemistry and thus their capability to transfer liquid.

Given the data collected, we suggest a mechanism for HH behaviour of the two model organisms *P. putida* and *P. ultimum*. Movement of liquid by *P. ultimum* hyphae across unsaturated environments created a scaffold of interconnected liquid patches that can be accessed by swimming *P. putida*. The presence of *P. putida* promotes liquid patch fusion, thus increasing the space available for bacterial movement.

Overall, we successfully developed a new tool for the direct observation and quantitative study of HHs, which allowed us to identify the role of liquid architecture on the functioning of HHs. While a lot remains to be uncovered, the HHD will be an invaluable tool to the advancement of the field. Future studies will utilize it to shed light on the mechanistic interactions between a variety of mycelial species and their micro-interactors. The HHD will allow us to increase our knowledge of true FHs by providing a tool to visualize ecologically relevant fungal-bacterial interactors. A good candidate would be *C. cinerea*, a dung saprophytic fungus thriving in organic-rich degrading materials such as dung or compost, that has been shown to interact with bacterial species *P. putida, Cupriavidus necator*, or *Cupriavidus oxalaticus* (Junier et al. 2021), as well as *Fusarium oxysporum*, which forms highways with *Mycobacterium frederiksbergense* (Kohlmeier et al. 2005). Some known FH-forming fungal species, such as *T. rossicum* and *F. gramineaurum* (Bouchard et al. 2026), are already known to grow well within non-saturated microfluidic channels (Gimeno et al. 2021). Furthermore, the use of fluorescently tagged fungal and fungal-like organisms, such as *C. cinerea* dTomato (Stanley et al. 2014) or *Phytophthora palmivora*-YFP (Rey et al. 2015), in combination with the techniques already proposed here will allow better quantification of mycelial network architecture with parameters such as hyphal density, hyphal length and network branching. Alternatively, fluorescent dyes can be used to investigate interaction with ecologically relevant soil microorganisms that are not amenable to transformation (Richter et al. 2023). Furthermore, the ability to sample from both the inlet and the outlet will allow further investigation of chemical and gene expression changes in the system as well as assessing network selectivity of bacterial species and more complex hyphal networks comprising multiple fungal species.

## Supplementary Material

uqag035_Supplemental_Files

## Data Availability

The data underlying this article are available on Zenodo at https://doi.org/10.5281/zenodo.20071448 (Moratto et al. 2026). All macros used to conduct semiautomated image analyses are included in the Supplementary material.
