# Supplementary material for: A microfluidic platform reveals the role of liquid movement on the dispersal of Pseudomonas putida via Pythium ultimum hyphal highways
Source: Microlife. 2026 Sep 21;7:uqag035. doi: 10.1093/femsml/uqag035 (PMC13637486; doi:10.1093/femsml/uqag035)
Supplement: Supplementary file 1 [file uqag035_supplemental_files.zip › Moratto_HyphalHighways_SI.pdf]

A microfluidic platform reveals the role of liquid movement on the dispersal of *Pseudomonas putida* via *Pythium ultimum* hyphal highways

Eleonora Moratto*[1], Emily Masters-Clark*[1], Amelia J. Clark[1], Aliya Pachmann[1], Cameron W. M. Murphy[2,3], Saskia Bindshedler[4], Pilar Junier[4], Claire E. Stanley[1, a)]

* These authors have contributed equally

[1] Department of Bioengineering, Imperial College London, London, SW7 2AZ, UK

[2] Department of Civil and Environmental Engineering, Imperial College London, London, SW7 2AZ, UK

[3] Centre for Environment and Life Sciences, CSIRO Land and Water, Private Bag No 5, Wembley, WA 6913, Australia

[4] Laboratory of Microbiology, University of Neuchâtel, Neuchâtel, CH-2000, Switzerland

[a)] Author to whom correspondence should be addressed: claire.stanley@imperial.ac.uk

Supplementary material

Supplementary figures

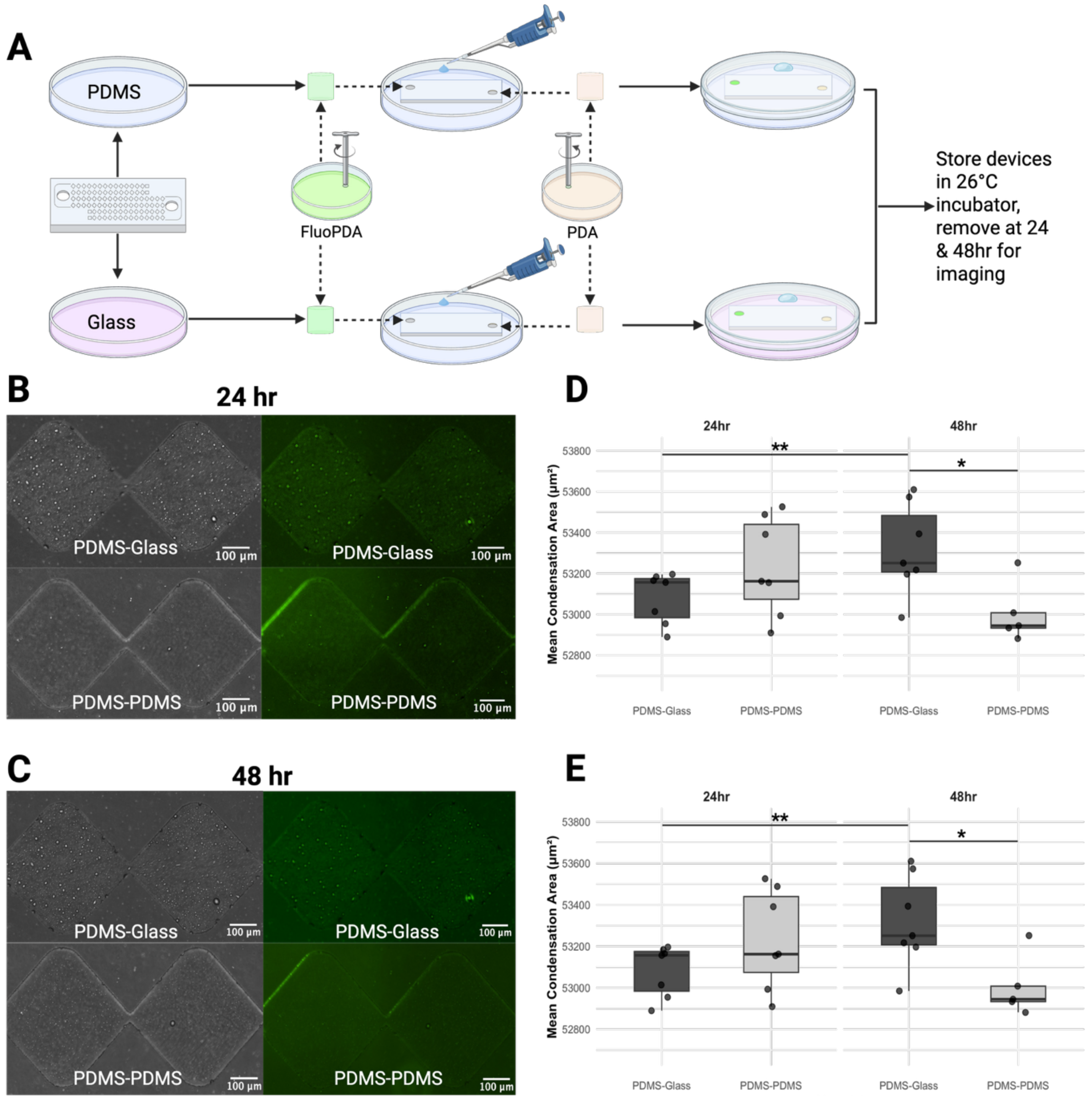

Supplementary Figure 1: PDMS spin-coating of glass petri dishes decreases condensation. Panel A illustrates the protocol for investigating the effect of bonding PDMS slabs to either a PDMS or glass base on condensation. To summarise, the cut and washed FFI PDMS slab is bonded to either a glass-bottomed Petri dish with or without a spin-coated layer of PDMS. Then, two agar plugs are placed into the inlets – one plug of FluoPDA (PDA with 0.66 µM fluorescein) and one plug of non-fluorescent PDA. 100 µl of Milli-Q water is added to the petri dish before sealing it with parafilm and storing the devices in an incubator at 26 °C, removing them at 24 and 48 hours post-inoculation for imaging. In the second interaction channel of the FFI device, images of Diamonds 1 and 2 bonded to either PDMS or glass were captured at 24 hours (B) and 48 hours (C) using phase contrast and fluorescence microscopy. Boxplots of the mean condensation area (µm^2) of PDMS-glass and PDMS-PDMS devices calculated using phase contrast (D) and fluorescence (E) images showed nearly identical results. Statistical analysis on both phase contrast and fluorescence microscopy datasets yielded the same results. The mean condensation area was analysed using the non-parametric Brunner-Munzel test, appropriate for small, unequal, and heterodaisic datasets. Results showed comparable performance between the setups at 24 hours ($p = 0.4375$), but at 48 hours, the PDMS-glass devices demonstrated significantly more condensation than PDMS-PDMS devices

(p = 0.02785). Notably, PDMS-glass exhibited a substantial rise in condensation area from 24 to 48 hours (p = 0.00732), while PDMS-PDMS showed no significant change (p = 0.0949).

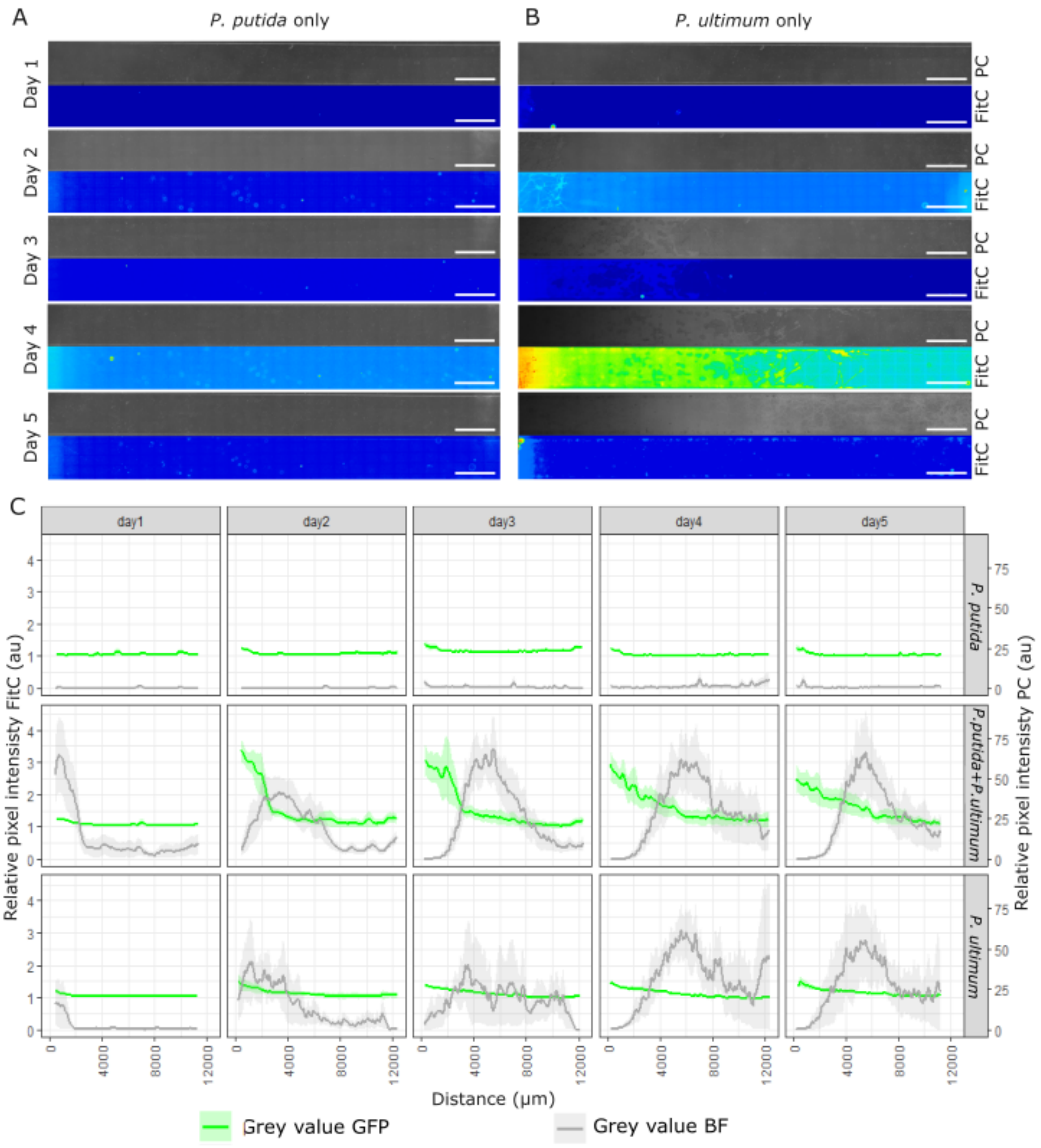

Supplementary Figure 2: A) & B) Representative images of the HHD channel inoculated with *P. putida* only (A) and *P. ultimum* only (B) 1 to 5 dpmi. Bacteria were inoculated on day 1 after imaging. Top panels were acquired using phase contrast (PC) and the bottom panels using FITC filter, enabling the visualisation of the mycelium and bacteria, respectively. The FITC image is displayed with a 16-colour LUT look-up table with blue representing the low pixel intensity and red representing high pixel intensity. Scale bars, 1 mm. All images depict the inside of the main observation channel only, measuring 12.5 mm in length (Fig. 1C). The images are oriented with the inlet on the left-hand side to match the orientation x-axis of each graph in panel C. **C)** Quantification of the relative pixel intensity of the FITC (green line) and PC (grey line) images along the 12.5 mm microchannel (see Supplementary Macro 1 for details). Images are taken each day of devices inoculated with *P. ultimum* and *P. putida*, *P. putida* only and *P. ultimum* only. The solid line represents the mean value, and the corresponding shaded area, the standard error.

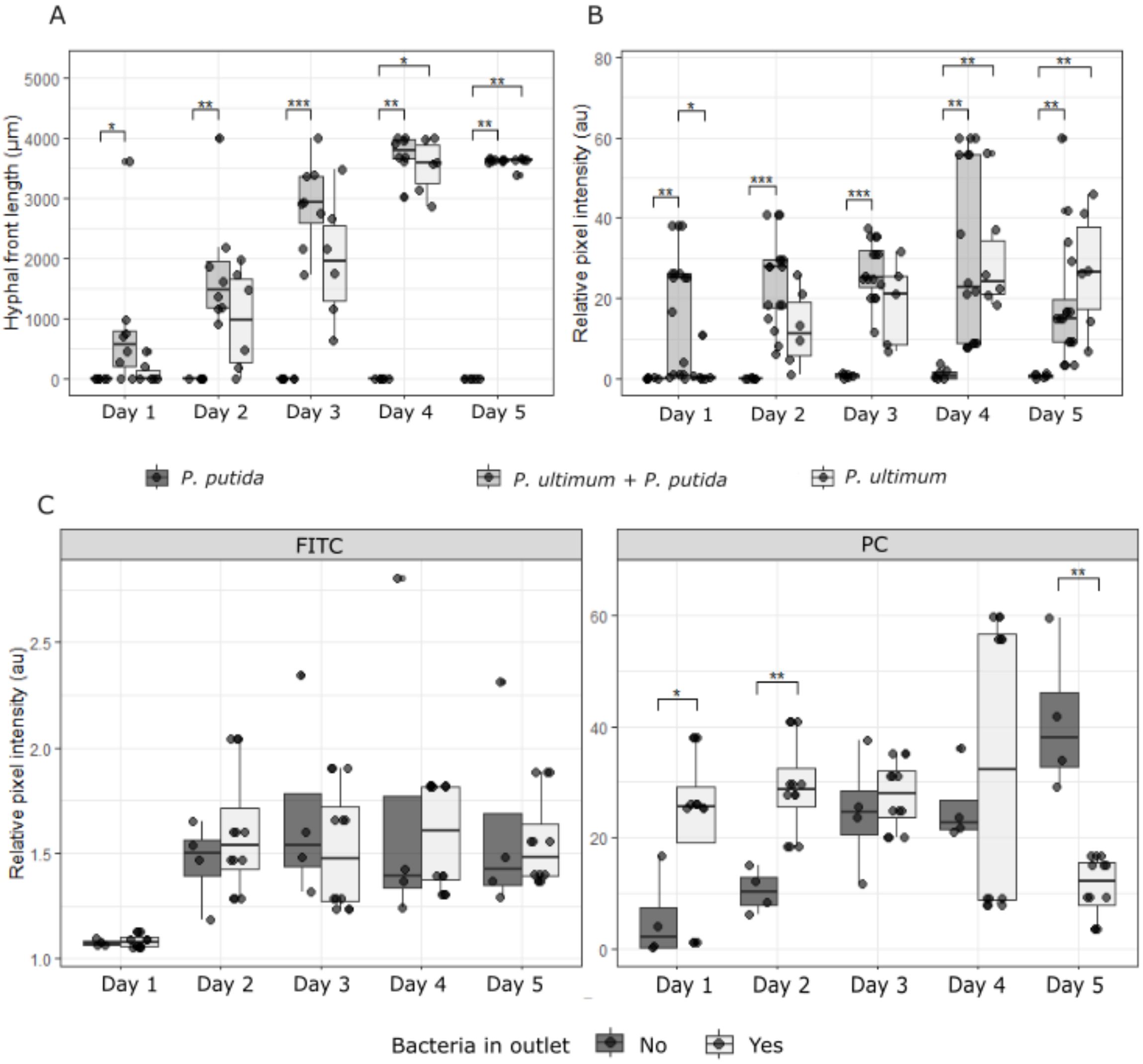

Supplementary Figure 3: *P. putida* KT2440::gfp has no impact on *P. ultimum* growth. **A)** Comparison of hyphal front length from the HHDs inoculated with (i) *P. putida* only (dark grey, n = 6), (ii) *P. ultimum* only (grey, n = 6), (iii) both *P. ultimum* and *P. putida* (light grey, n = 8) each day. A Dunn's post-hoc test was run to identify pairwise differences, and significance is indicated by an asterisk (* $p \leq 0.05$; ** $p \leq 0.01$; *** $p \leq 0.001$). **B)** Comparison of average relative pixel intensity across the channel for HHDs inoculated with (i) *P. putida* only (dark grey, n = 6), (ii) *P. ultimum* only (grey, n = 6), (iii) both *P. ultimum* and *P. putida* (light grey, n = 8) each day. A Dunn's post-hoc test was run to identify pairwise differences, and significance is indicated by an asterisk (* $p \leq 0.05$; ** $p \leq 0.01$; *** $p \leq 0.001$). **C)** Comparison of FITC and PC average relative pixel intensity across the channel for FHDs inoculated with both *P. ultimum* and *P. putida* (n = 8), split based on the presence (light grey) or absence (dark grey) of bacteria in the outlet. A Dunn's post-hoc test was run to identify pairwise differences, and significance is indicated by an asterisk (* $p \leq 0.05$; ** $p \leq 0.01$).

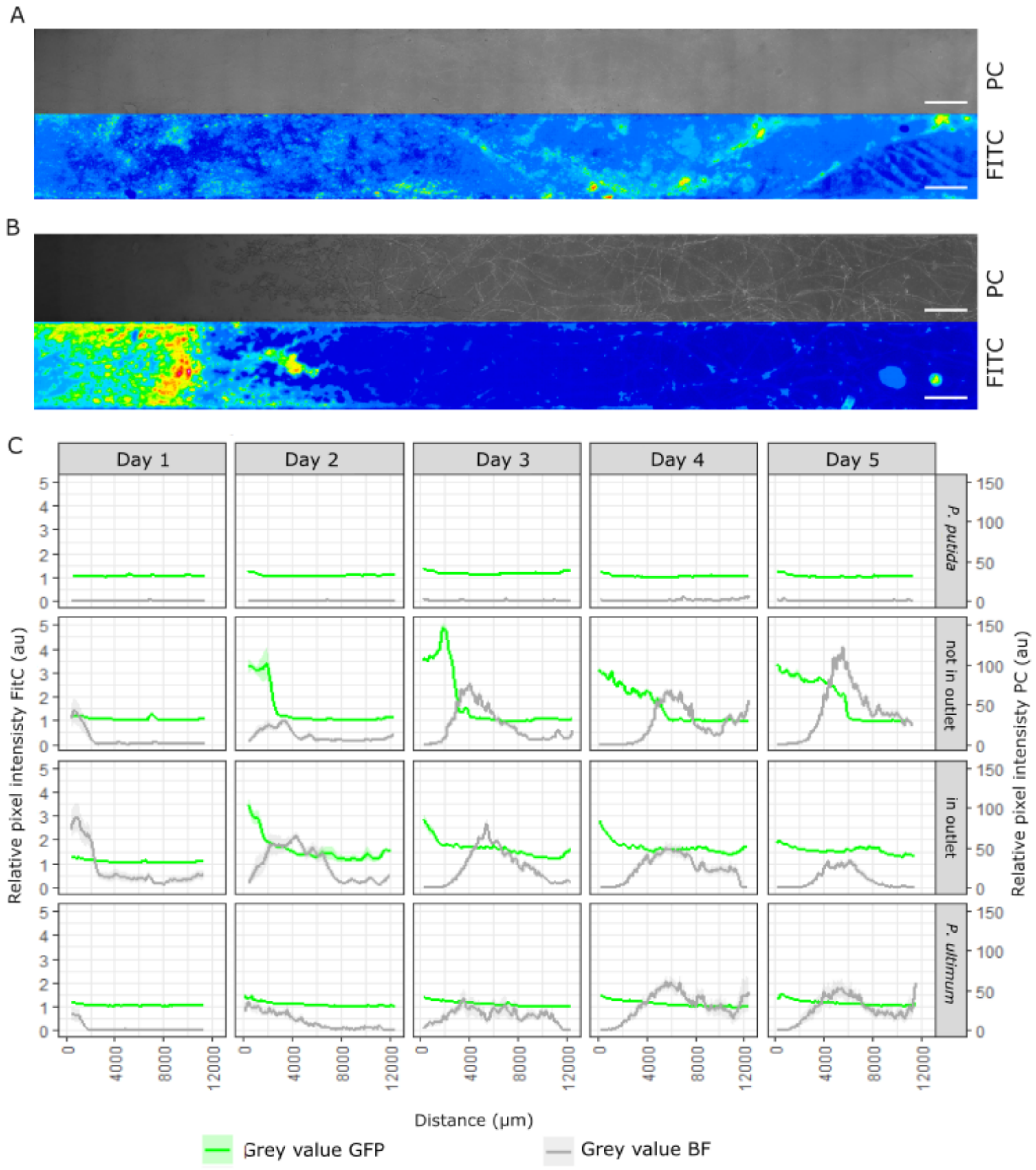

Supplementary Figure 4: A) & B) Representative images of the HHD channel containing both *P. putida* and *P. ultimum* at 5 dpmi. The bacteria were recovered in the outlet in (A) but not in (B). Top panels were acquired using phase contrast (PC) and the bottom panels using FITC filter, enabling the visualisation of the mycelium and bacteria, respectively. The FITC image is displayed with a 16-colour LUT look-up table with blue representing the low pixel intensity and red representing high pixel intensity. Scale bars, 500 µm. All images depict the inside of the main observation channel only, measuring 12.5 mm in length (Fig. 1C). The images are oriented with the inlet on the left-hand side to match the orientation of the x-axis of each graph in panel C. **C)** Quantification of the relative pixel intensity of the FITC (green line) and PC (grey line) images along the 12.5 mm microchannel (see Supplementary Macro 1 for details**).** Images are taken each day of devices inoculated with *P. ultimum* and *P. putida* where bacteria were present in the outlet (n = 4), *P. ultimum* and *P. putida* where bacteria were absent from the outlet (n = 4), *P. putida* only (n = 6), and *P. ultimum* only (n = 6). The solid line represents the mean value, and the corresponding shaded area, the standard error.

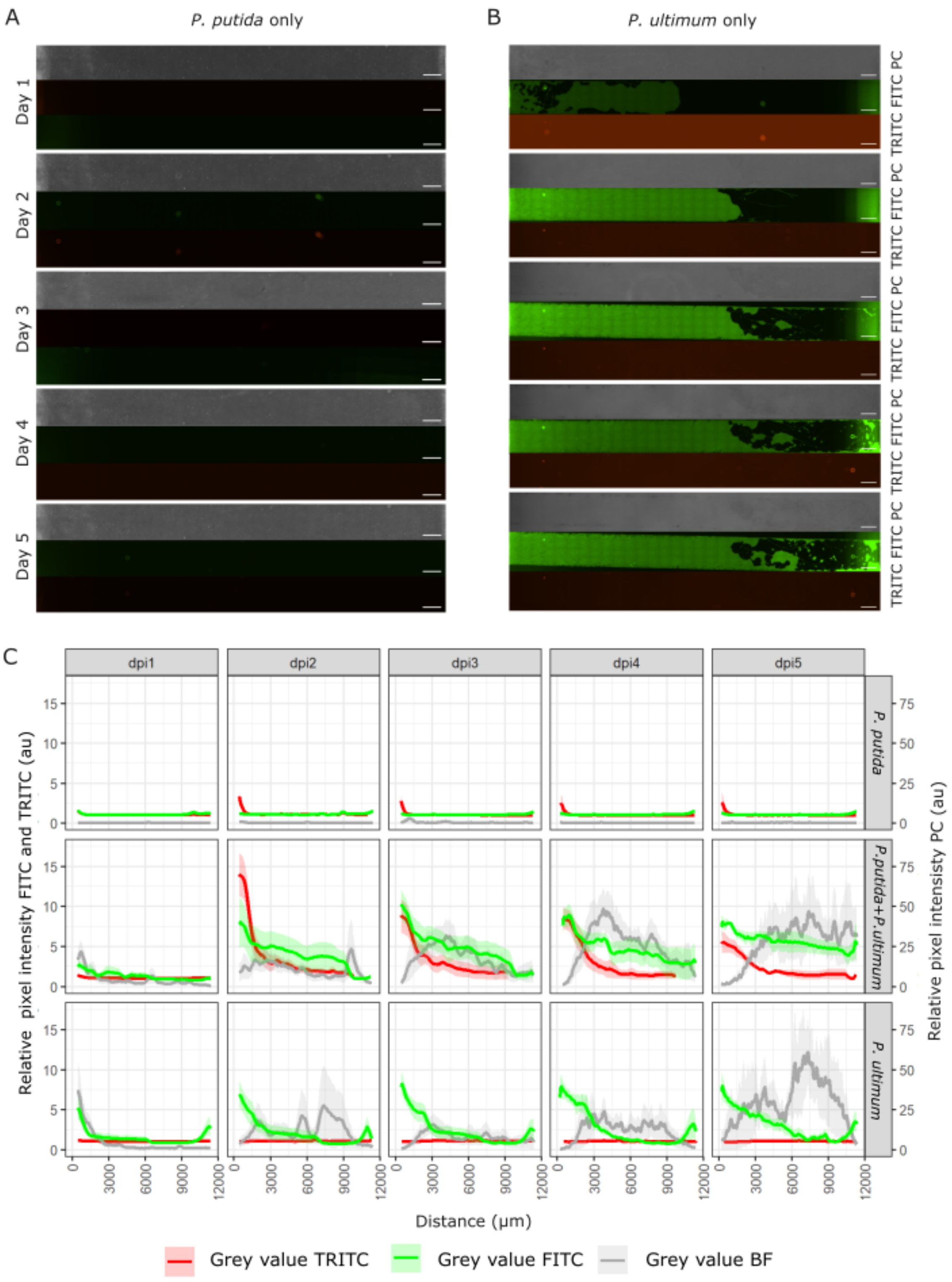

Supplementary Figure 5: A) & B) Representative images of the FHD channel inoculated with *P. putida* only (A) and *P. ultimum* only (B) 1 to 5 dpmi. Bacteria were inoculated on day 1 after imaging. Top panels were acquired using phase contrast (PC), the middle panels using FITC filter and the bottom panels using the TRITC filter, enabling the visualisation of the mycelium, the liquid and bacteria, respectively. Scale bars, 500 µm. All images depict the inside of the main observation channel only, measuring 12.5 mm in length (Fig. 1C). The images are oriented with the inlet on the left-hand side to match the orientation of the x-axis of each graph in panel C. **C)** Quantification of the relative pixel intensity of the TRITC (red line), FITC (green line) and PC (grey line) images along the 12.5 mm microchannel (see Supplementary Macro 4 for details). Images are taken each day of devices

inoculated with *P. ultimum* and *P. putida*, *P. putida* only and *P. ultimum* only. The solid line represents the mean value, and the corresponding shaded area, the standard error.

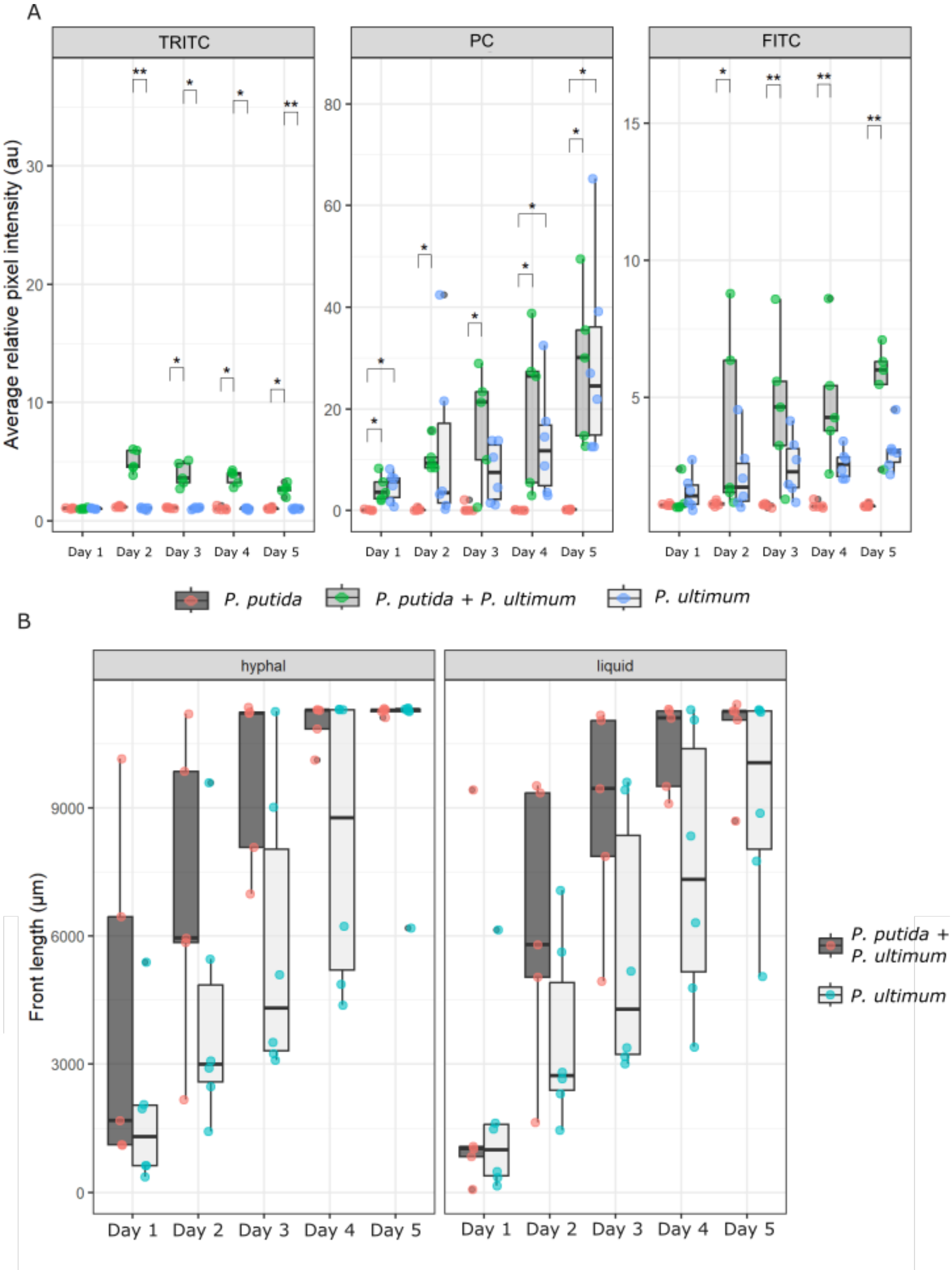

Supplementary Figure 6: *P. putida* F1 mScarlet-I has no impact on *P. ultimum* growth or liquid transport. **A)** Comparison of average relative pixel intensity across the channel for HHDs inoculated with (i) *P. putida* only (dark grey, red, n = 5), (ii) *P. ultimum* only (light grey, blue, n = 5), (iii) both *P. ultimum* and *P. putida* (grey, n = 5) each day. A Dunn's post-hoc test was run to identify pairwise differences, and significance is indicated by an asterisk (* $p \leq 0.05$; ** $p \leq 0.01$). **B)** Comparison of front length from the HHDs inoculated with (i) *P. ultimum* only

(dark grey, red, n = 5), (ii) both *P. ultimum* and *P. putida* (light grey, blue, n = 5) each day. From left to right, panels show hyphal front and liquid front. A Dunn's post-hoc test was run to identify pairwise differences, and no significant differences were found.

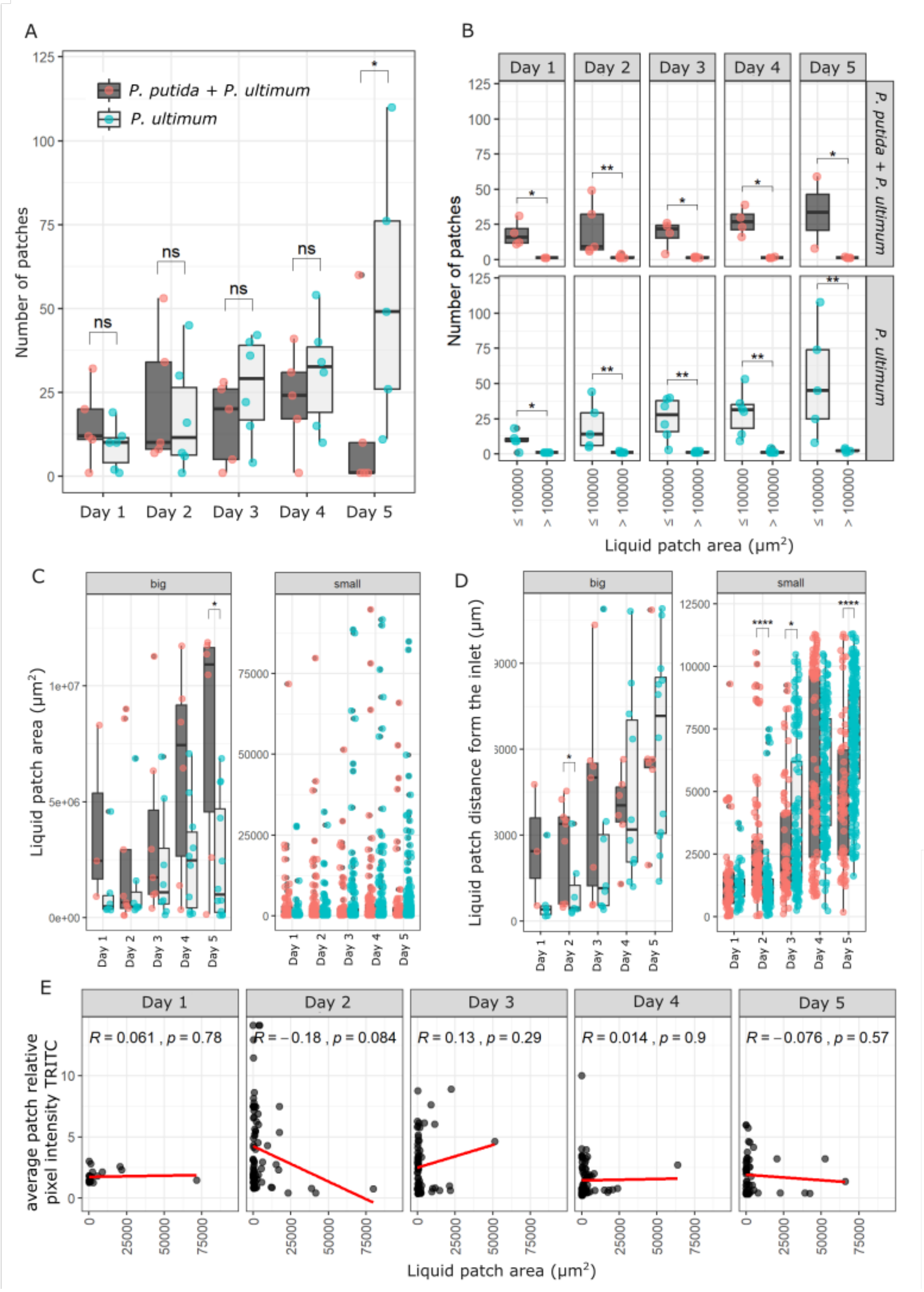

Supplementary Figure 7: *P. putida* effect on liquid patches. A) Comparison of the number of liquid patches in the FHDs inoculated with *P. ultimum* only (dark grey, n = 6) and both *P. ultimum* and *P. putida* (light grey, n = 5) each day. A Dunn's post-hoc test was run to identify pairwise differences, and no significance was found. **B)**

Number of liquid patches in the HHDs inoculated based on two bins defined by area, larger and smaller than 1×10^5 μm^2. Each point represents one device for the specified day inoculated with *P. ultimum* only (blue, n = 6) and *P. ultimum* and *P. putida* (red, n = 5). We find significantly more small patches than large patches. Significance is indicated by an asterisk (Dunn's post-hoc test, * $p \leq 0.05$; ** $p \leq 0.01$). **C)** Liquid patch area each day. The data is split into big ($\leq 1\times10^5$) and small ($>1\times10^5$) patches. Each point represents one patch in an HHD for the specified day inoculated with *P. ultimum* only (blue, n = 6) and *P. ultimum* and *P. putida* (red, n = 5). We show a significant difference in big patches area on day 5 (Dunn's post-hoc test, * $p \leq 0.05$). **D)** Liquid patch distance from the inlet each day. The data is split into big ($\leq 1\times10^5$) and small ($>1\times10^5$) patches. Each point represents one liquid patch in an HHD inoculated with *P. ultimum* only (blue, n = 6) and *P. ultimum* and *P. putida* (red, n = 5). Significant differences are shown with asterisks (Dunn's post-hoc test, * $p \leq 0.05$, **** $p \leq 0.0005$). **E)** Linear regression analysis of average patch relative pixel intensity for the TRITC channel against patch area. Each dot represents one liquid patch. We find no correlation between pixel intensity and liquid patch area.

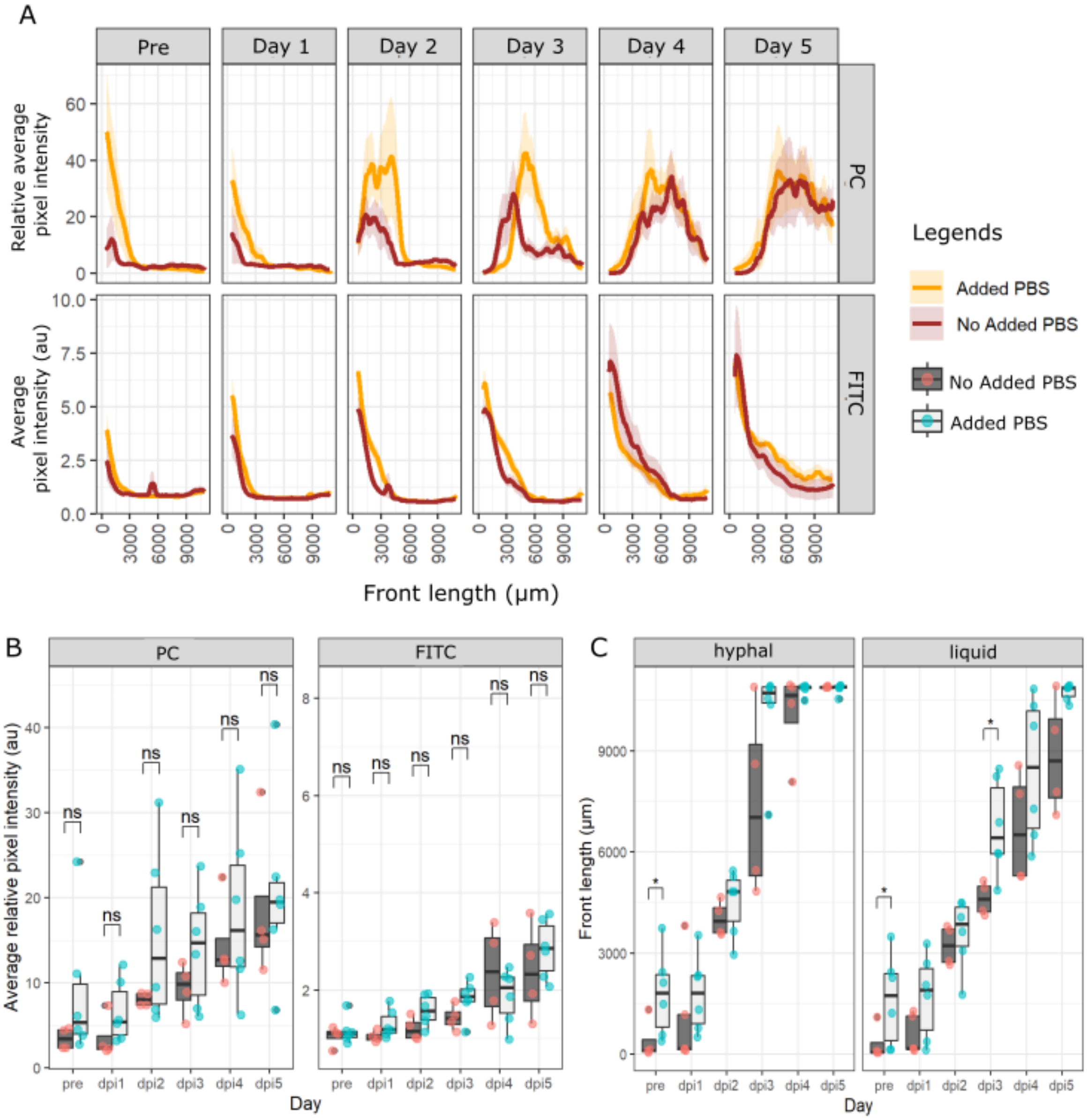

Supplementary Figure 8: Addition of 10 µL PBS does not affect fungal biomass or liquid distribution. A) Quantification of the relative pixel intensity of the PC and FITC images (Supplementary Macro 1) along the channel each day of devices inoculated with *P. ultimum* with added 10 µL PBS (orange) and *P. ultimum* without added 10 µL of PBS. The solid line represents the mean value and the corresponding shaded area, the standard error (n = 6). **B)** Comparison of average relative pixel intensity across the channel for HHDs inoculated with *P. ultimum*

without added 10 µL PBS (dark grey, red, n = 5) or *P. ultimum* with added 10 µL PBS (light grey, blue, n = 5) each day. A Dunn's post-hoc test was run to identify pairwise differences, and no significant differences were found. **C)** Comparison of front length from the HHDs inoculated with *P. ultimum* without added 10 µL PBS (dark grey, red, n = 5) or *P. ultimum* with added 10 µL PBS (light grey, blue, n = 5) each day. From left to right, panels show hyphal front and liquid front. Significant differences are shown with asterisks (Dunn's post-hoc test, * $p \leq 0.05$).

Supplementary Tables

Supplementary Table 1: List and origin of microorganisms used

Microorganism name	Origin
Pythium ultimum M194	Laboratory of Microbiology (University of Neuchâtel, Switzerland)
Pseudomonas putida strain KT2440::gfp	Department of Biotechnology and Biomedicine (Technical University of Denmark, Denmark)
Pseudomonas putida F1 mScarlet-I (pMRE135)	Generated for this publication (Imperial College London, United Kingdom)
Pseudomonas putida F1 WT	College of Biological Sciences (UC Davis, United States)

Supplementary Table 2: Microscopy equipment and settings used to obtain microscopy images

Equipment	Eclipse Ti-U (Nikon)
Objective	Air immersed
Camera	Retiga R1 CCD camera (Qimaging)
Stage	Prior Scan III motorized stage
Illumination	High-power light emitting diode (LED) (Omicron-Laserage Laserprodukte GmbH)
Filter Sets	**TRITC filter set:**<br>Beamsplitter: 565 nm (AHF Analysentechnik AG)<br>Emission filter: 605/70 nm (570-640 nm) (AHF Analysentechnik AG)<br>**FITC filter set:**<br>Beamsplitter: 495 nm (AHF Analysentechnik AG)<br>Emission filter: 525/50 nm (500-550 nm) (AHF Analysentechnik AG)

Supplementary Videos

Supplementary Video 1: Real-time video of the HHD channel inoculated with *Pythium ultimum* and *Pseudomonas putida*-GFP. The video was recorded in real time at 40x magnification using phase contrast microscopy first and switching to the FITC filter thereafter. *P. putida* swims within liquid patches formed around *P. ultimum* hyphae. The bacteria concentrate at the perimeter of the liquid patch and move along a single hypha surrounded by a fine liquid film.

Supplementary Video 2: Real-time video of the HHD channel inoculated with *Pythium ultimum* and *Pseudomonas putida*-GFP. The video was recorded in real time at 40x magnification using phase contrast microscopy first and switching to the FITC filter thereafter. *P. putida* moves within liquid patches along *P. ultimum* hyphae. Bacteria are absent from discontiguous liquid patches.

Supplementary Video 3: Real-time video of the HHD channel inoculated with *Pythium ultimum* and *Pseudomonas putida*-GFP. The video was recorded in real time at 40x magnification using phase contrast microscopy first and switching to the FITC filter thereafter. *P. putida* actively moves between contiguous liquid patches along hyphae and pulsing liquid films.

Supplementary Video 4: Real-time video of the HHD channel inoculated with *Pythium ultimum* and *Pseudomonas putida*-GFP. The video was recorded in real time at 40x magnification using phase contrast microscopy first and

switching to the FITC filter thereafter. High concentration of *P. putida* being trapped by hyphae within a small section of liquid film.

Supplementary Video 5: Real-time video of the HHD channel inoculated with *Pythium ultimum* and *Pseudomonas putida*-GFP. The video was recorded in real time at 40x magnification using phase contrast microscopy first and switching to the FITC filter thereafter. Pulsing liquid films cause rapid movement of bacteria and inversion of the bacterial flow along *P. ultimum* hyphae.

Supplementary Video 6: Real-time video of the HHD channel inoculated with *Pythium ultimum* and *Pseudomonas putida*-MScarlet. The video was recorded in real time at 20x magnification, using phase contrast microscopy first, then switching to the FITC filter to visualise fluorescein-stained liquid, and then the TRITC filter to visualise the MScarlet-tagged bacteria. Bacteria were seen to move bidirectionally along hyphae extending from colonised liquid patches.

Supplementary Methods

Construction of the chemotactic **P. putida** *F1 mScarlet-I (pMRE135) strain.*

The *P. putida F1* WT strain was modified by incorporating a mScarlet-I (pMRE135) plasmid inside the *P. putida* F1 WT strain. A single *P. putida F1* WT colony was selected from a nutrient agar (Oxoid) plate, which had been incubated overnight (Thermo Scientific MaxQTM 6000) at 30 °C. The *P. putida* F1 WT strain was initially streaked from a -80 °C (New Brunswick Scientific U535) glycerol stock (50% autoclaved Milli-Q© and 50% 0.2 µm filtered (Corning©) glycerol ≥ 99.5% (Fisher BioReagentsTM). This colony was subsequently incubated overnight in 5 ml lysogeny broth (LB) (Miller, Fisher BioReagentsTM) within a 14 ml culture tube (Starlab), shaking at 250 rpm and 30 °C (Luu et al., 2013).

The overnight culture was then centrifuged; 4000 x g (Eppendorf Centrifuge 5810 R), pelleted and washed twice at room temperature (21 °C), for 10 minutes in 0.2 µm vacuum filtered 300 mM sucrose (Sigma). This method of making electrocompetent cells is adapted from (Choi et al., 2006). This sucrose was dissolved in H_2O using a magnetic stirrer (FisherbrandTM). All centrifugations occur at room temperature, unless otherwise stated. The electrocompetent cell culture was then concentrated by centrifugation, and 1 mL was pipetted into a 2 ml Eppendorf, with vortexing (Corning© LSE). This suspension was then centrifuged for 8 minutes at 2000 x g using an Eppendorf 5453 MiniSpin table-top centrifuge. The supernatant was then discarded and the pellet was resuspended in 0.5 ml of 300 mM sucrose solution (Choi et al., 2006).

An *E. coli* S17-1 λpir strain carrying the mScarlet-I (pMRE135) plasmid colony was selected from a LB agar (Miller, Supelco©) plate dosed with 50 µg/ml kanamycin and 25 µg/ml chloramphenicol, which had been had been incubated overnight at 30 °C. Visual confirmation of the fluorescence was achieved using a UV light box (Alpha Innotech). A fluorescent colony was incubated overnight (16 hours) in 5 ml.

LB within a 14 ml culture tube at 37 °C and 250 rpm. It was then dosed with 50 µg/ml kanamycin and chloramphenicol 25 µg/ml, to maintain the presence of the fluorescent mScarlet-I plasmid (Addgene, Bacterial resistance: kanamycin, Backbone selectable markers: chloramphenicol #118489) (Bindels et al., 2017). The colony was then pelleted twice in H_2O at 4000 x g for 10 minutes. AQIA prep Spin Miniprep kit from Qiagen was then used to extract the plasmid to a concentration of 50 ng/ml, which was measured on a Thermo ScientificTM NanoDrop 1000 spectrophotometer. A 100 µl aliquot of the concentrated electrocompetent *P. putida* F1 WT cell suspension was then pipetted into a 2 ml electroporation cuvette (Bio-Rad) after vortexing, along with 1.25 µl of the mScarlet-I pMRE135 plasmid DNA and electroporated (2 ml Bio-Rad MicroPulsar, Standard *E. coli*. 2 set-up; 2.5 kV) (Choi et al., 2006). One ml of

autoclaved LB was immediately added, and cells were incubated in a spinning incubator at 250 rpm and 30 °C for 1 hour. This mixture was then centrifuged for 8 minutes at 2000 x g and spread on an LB plate using a Microspec E6 Spreader (Fisher ScientificTM). The plate was also dosed with 50 µg/ml kanamycin and 25 µg/ml chloramphenicol and incubated at 33.5 °C (ColeParmerTM StuartTM SI-200) overnight.

Plates were screened for positively fluorescence colonies using a UV light box, then 82 overnight cultures were prepared in 5 ml LB within a 14 ml culture tube dosed with 50 µg/ml kanamycin and 25 µg/ml chloramphenicol at 30 °C to make glycerol stocks. The new *P. putida* F1 mScarlet-I (pMRE135) strain was then placed in the -80 °C freezer in glycerol stock using the same ratios previously described.

Testing the effect of a PDMS base on condensation

To test the ability of condensation to form on different surfaces, we created FFI devices (Gimeno et al., 2021) by bonding PDMS slabs to glass-bottomed Petri dishes either with or without a PDMS coating (see *Device design and fabrication* and Supplementary Figure 1). To observe condensation dynamics independent of biotic factors, methods were adapted from Clark et al.'s protocols for visualising liquid redistribution within the same device (Clark et al., 2024). FFI devices were inoculated with (i) a PDA plug and (ii) a PDA plug supplemented with 0.66 µM filter-sterilised fluorescein (Clark et al., 2024) before being placed in an incubator at 26 °C. Devices were removed at 24 hours post-inoculation (hpi) and 48 hpi for phase contrast (PC) and fluorescence microscopy imaging using a Nikon Eclipse Ti-2 inverted microscope. Microscope images were analysed in FIJI (Schindelin et al., 2012). Images were converted to 8-bit .tiff files, before performing Renyi's entropy-based thresholding and condensation area within the channels was measured and recorded (Supplementary Macros 2 & 3). Statistical analysis was performed in RStudio using the Dplyr, ggplot2, and brunnermunzel packages. The non-parametric Brunner Munzel test was used for significance testing between timepoints and device conditions.

Testing the effect of adding 10 µL of PBS on liquid distribution

To assess whether the addition of 10 µL of PBS to the bacterial-fungal condition affected growth rate and channel saturation, a control experiment was conducted on the FHD platform, following the same device manufacturing, microbial culture conditions, and inoculation procedures as detailed in the materials and methods of this paper. All devices were inoculated with a plug of *P. ultimum* grown on PDA with fluorescein, and a plug of PDA containing fluorescein was placed at the outlet. To prevent desiccation, 100 µL of water was added outside the chip in the PDMS-coated dish. After 24 h, all devices were imaged. Subsequently, 10 µL of PBS was added to half of the devices, a new fluorescein-containing PDA plug was placed on top, and the devices were resealed. For the control devices without PBS, a fluorescent-containing PDA plug was placed at the inlet, and they were resealed. No bacteria were added to any of the devices. All devices were imaged again post-resealing and periodically until day 5. The resulting time series were analysed using Fiji.

References

Bindels, D.S., Haarbosch, L., Van Weeren, L., Postma, M., Wiese, K.E., Mastop, M., Aumonier, S., Gotthard, G., Royant, A., and Hink, M.A. (2017). mScarlet: a bright monomeric red fluorescent protein for cellular imaging. *Nature Methods*. 14, 53–56.

Choi, K.-H., and Schweizer, H.P. (2006). Mini-Tn7 insertion in bacteria with single attTn7 sites: example *Pseudomonas aeruginosa*. *Nature Protocols.* 1, 153–161

Luu, R.A., Schneider, B.J., Ho, C.C., Nesteryuk, V., Ngwesse, S.E., Liu, X., Parales, J.V., Ditty, J.L., and Parales, R.E. (2013). Taxis of *Pseudomonas putida* F1 toward phenylacetic acid is mediated by the energy taxis receptor Aer2. *Applied and Environmental Microbiology*. 79, 2416–2423.

Supplementary Macros

Here, we report all macros used to conduct semiautomated image analyses within the software ImageJ. All are designed to be opened in ImageJ and run in their entirety, but do require user intervention, which is indicated by instructions appearing on screen.

Macro 1 is written in ImageJ Macro language (IJM). The macro is designed to analyse a raw .nd2 file containing the tiled phase contrast image in channel 1 and a fluorescent FITC image in channel 2. The macro outputs three .csv files containing the raw average pixel intensity measured along the device in each channel and a background value for FITC, respectively.

Macros 2 and 3 are written in Jython, a Java implementation of Python. Microscopy files are pre-processed into .tiff hyperstacks with two channels; phase contrast and FITC, and saved into a folder for batch processing with Macro 2 and 3. Macro 2 and Macro 3 are executed in sequential order. Macro 2 is designed to analyse .tiff hyperstacks containing two channels: phase contrast and FITC. It uses user-drawn lines to correct any skew in the image and employs a user-defined bounding box to geometrically transform the region of interest (ROI) pattern onto the image, outlining the channels. The transformed ROI patterns and the deskewed images are then saved into their respective folders as .zip files (for the ROI) and .tiff files (for the two channels). Macro C requires the user to select a folder containing the deskewed images of the channel of interest, along with the .zip files of the transformed ROIs. After selection, the macro thresholds the images using Renyi's entropy thresholding method. It then calculates the total area of each ROI, the area within the ROI that registers above the threshold, and the percentage of the ROI which presents a signal (% signal coverage). Note: this macro requires .tiff microscopy files with scale information stored in their metadata.

Macro 4 is an extension of Macro 1. The macro is designed to analyse a raw .nd2 file containing the tiled phase contrast image in channel 1, a fluorescent FITC image in channel 2 and a fluorescent TRITC image in channel 3. The macro outputs four .csv files containing the raw average pixel intensity measured along the device in each channel and a background value for FITC and TRITC, respectively.

Macro 5 is written in ImageJ Macro language (IJM) to analyse a raw .nd2 file containing the tiled phase contrast image in channel 1 and a fluorescent TRITC image in channel 2 and a fluorescent FITC image in channel 3. The macro outputs a .csv file containing the area and position of liquid patches in the channel based on FITC and the average TRITC pixel intensity in each patch.

Supplementary Macro 1: HHD image analysis for hyphal and bacterial quantification

```
//Set line width to channel width
run("Set Measurements...", "area mean min perimeter redirect=None decimal=3");
run("Line Width...", "line=3000");
setTool("line");

//Get directory and Image name
dir1 = getDirectory("image");
imageName = getTitle();
// Remove the file extension from the image name
dotIndex = indexOf(imageName, ".");
if (dotIndex != -1) {
   imageName = substring(imageName, 0, dotIndex);
}

//Duplicate image
run("Duplicate...", "duplicate");
rename("Image");

//Draw line
waitForUser("Draw line along the channel, then hit OK");
roiManager("Add");

//Split channels
run("Split Channels");

//Measure line green
selectImage("C2-Image");
roiManager("Select", 0);
run("Plot Profile");
```

```
Plot.getValues(xValues, yValues);
csvFilePath = dir1 + imageName + "_bacteria.csv";
file = File.open(csvFilePath);
print(file, "Distance_microns,Gray_Value");
for (i = 0; i < xValues.length; i++) {
   print(file, xValues[i] + "," + yValues[i]);
}
File.close(file);
//print("Plot profile data saved as: " + csvFilePath);
selectImage("Plot of C2-Image");
run("Close");

//Make hyphae into binary
selectImage("C1-Image");
run("Gaussian Blur...", "sigma=0.7 stack");
run("8-bit");
run("Sharpen");
run("Subtract Background...", "rolling=30");
waitForUser("adjust threshold", "test the threshold parameters in the window that next appears.");
run("Threshold...");
waitForUser("Click Apply then click OK");
run("Dilate");
run("Analyze Particles...", "size=100-Infinity pixel circularity=0.00-0.15 show=Masks");

//Measure line hyphae
selectWindow("Mask of C1-Image");
roiManager("Select", 0);
run("Plot Profile");
Plot.getValues(xValues, yValues);
csvFilePathf = dir1 + imageName + "_fungi.csv";
```

```
filef = File.open(csvFilePathf);
print(filef, "Distance_microns,Gray_Value");
for (i = 0; i < xValues.length; i++) {
    print(filef, xValues[i] + "," + yValues[i]);
}
File.close(filef);
//waitForUser("Save plot data, then press ok");
selectImage("Plot of Mask of C1-Image");
run("Close");

//Measure green background
selectImage("C2-Image");
setTool("rectangle");
waitForUser("Draw Rectangle for background");
roiManager("Add");
roiManager("Select", 1);
roiManager("Measure");
saveAs("Results", dir1+imageName+"_bacteria_n.csv");

//Close everything
roiManager("Delete");
roiManager("Delete");
selectWindow("Results");
run("Close");
selectImage("Mask of C1-Image");
run("Close");
selectImage("C2-Image");
run("Close");
selectImage("C1-Image");
run("Close");
```

Supplementary Macro 2: Image processing & ROI alignment

```
# Macro 2 (Jython): Interactive Processing, ROI Alignment, Channel Splitting/Merging

from ij import IJ, ImagePlus
from ij.plugin.frame import RoiManager
from ij.gui import WaitForUserDialog, Roi, PolygonRoi, Line
import os, math
from ij.plugin import ChannelSplitter, RGBStackMerge
from ij.io import FileSaver, DirectoryChooser, OpenDialog

# ========================
# Helper Functions
# ========================

def transform_single_roi(roi, scale_x, scale_y, translate_x, translate_y):
    """
    Transform one ROI by scaling & translating. Adapt to correct type:
    - POLYGON, FREEROI, TRACED_ROI, POLYLINE, FREELINE, ANGLE => PolygonRoi
    - LINE => new Line
    - RECTANGLE => new rectangular Roi
    - Others => skip (return None)
    """
    roi_type = roi.getType()
    poly = roi.getFloatPolygon()
    roi_name = roi.getName() if roi.getName() else ""

    if roi_type in (Roi.POLYGON, Roi.FREEROI, Roi.TRACED_ROI, Roi.POLYLINE, 
Roi.FREELINE, Roi.ANGLE):
        # general polygon-based approach
        new_x = [(x * scale_x + translate_x) for x in poly.xpoints]
        new_y = [(y * scale_y + translate_y) for y in poly.ypoints]
```

```
        new_roi = PolygonRoi(new_x, new_y, len(new_x), roi_type)

    elif roi_type == Roi.RECTANGLE:
        # transform bounding rectangle
        bounds = roi.getBounds()
        new_x = bounds.x * scale_x + translate_x
        new_y = bounds.y * scale_y + translate_y
        new_w = bounds.width * scale_x
        new_h = bounds.height * scale_y
        new_roi = Roi(new_x, new_y, new_w, new_h)

    elif roi_type == Roi.LINE:
        # transform endpoints
        if len(poly.xpoints) == 2:
            x1 = poly.xpoints[0] * scale_x + translate_x
            y1 = poly.ypoints[0] * scale_y + translate_y
            x2 = poly.xpoints[1] * scale_x + translate_x
            y2 = poly.ypoints[1] * scale_y + translate_y
            new_roi = Line(x1, y1, x2, y2)
        else:
            # Possibly skip or handle differently
            return None
    else:
        # unsupported type => skip
        return None

    new_roi.setName(roi_name)
    return new_roi

def transform_rois(rois, ref_roi, user_roi):
    """
```

```
    Takes pattern rois, the reference bounding box ROI, and the user bounding box ROI.
    Returns the newly transformed ROIs.
    """
    ref_bounds = ref_roi.getBounds()
    user_bounds = user_roi.getBounds()

    # Compute scaling + translation
    scale_x = user_bounds.width / float(ref_bounds.width)
    scale_y = user_bounds.height / float(ref_bounds.height)
    translate_x = user_bounds.x - ref_bounds.x * scale_x
    translate_y = user_bounds.y - ref_bounds.y * scale_y

    transformed = []
    for roi in rois:
        new_roi = transform_single_roi(roi, scale_x, scale_y, translate_x, translate_y)
        if new_roi:
            transformed.append(new_roi)

    return transformed

def compute_rotation_angle(line_roi):
    """
    Computes rotation angle from a 2-point line ROI,
    returning an angle that avoids flipping the image upside down.
    """
    poly = line_roi.getFloatPolygon()
    x1, y1 = poly.xpoints[0], poly.ypoints[0]
    x2, y2 = poly.xpoints[1], poly.ypoints[1]
    angle = math.degrees(math.atan2(y2 - y1, x2 - x1))

    rotate_angle = -angle
```

```
    # Prevent over-rotation
    if rotate_angle > 90:
        rotate_angle -= 180
    elif rotate_angle < -90:
        rotate_angle += 180

    return rotate_angle

# ========================
# Main Script
# ========================

# Ensure ROI Manager Closed at Start
rm_existing = RoiManager.getInstance()
if rm_existing:
    rm_existing.close()

# Prompt for working directory containing converted .tiff s
dc = DirectoryChooser("Choose the Converted .tiff Directory")
work_dir = dc.getDirectory()

# Create subfolders if not present
output_dirs = ["Channel1", "Channel2", "Merged", "ROI"]
for odir in output_dirs:
    dir_path = os.path.join(work_dir, odir)
    if not os.path.exists(dir_path):
        os.makedirs(dir_path)

# Prompt for ROI pattern ZIP
roi_zip_path = OpenDialog("Choose ROI Pattern ZIP file").getPath()
rm_pattern = RoiManager()
```

```
rm_pattern.reset()
rm_pattern.runCommand("Open", roi_zip_path)

pattern_rois = [rm_pattern.getRoi(i) for i in range(rm_pattern.getCount())]
ref_bbox_roi = next((r for r in pattern_rois if r.getName() == "BoundingBox"), None)
if ref_bbox_roi is None:
    IJ.error("BoundingBox ROI not found. Exiting.")
    exit()

# Clear ROI Manager after verifying bounding box
rm_pattern.reset()

# ========================
# Process each TIF file
# ========================
for file_name in os.listdir(work_dir):
    if not file_name.lower().endswith(".tif"):
        continue

    file_path = os.path.join(work_dir, file_name)
    imp = IJ.openImage(file_path)
    if not imp:
        IJ.log("Failed to open: " + file_name)
        continue

    # Split into channels
    channels = ChannelSplitter.split(imp)
    ch1, ch2 = channels[0], channels[1]

    ch1.show()  # channel 1 is brightfield / interactive
    IJ.setTool("line")
```

```
# Initialize new ROI Manager for deskew
rm = RoiManager.getInstance()
if rm is None:
    rm = RoiManager()
rm.reset()

# Prompt for deskew line
WaitForUserDialog("Deskewing",
    "Draw a line along feature on channel 1. Add the line to ROI Manager, then click OK."
).show()

if rm.getCount() < 1 or rm.getRoi(0).getType() != Roi.LINE:
    IJ.log("Invalid deskew line. Skipping file: " + file_name)
    ch1.close()
    continue

# Compute & apply rotation
angle = compute_rotation_angle(rm.getRoi(0))
IJ.run(ch1, "Rotate...", "angle={} interpolation=Bilinear enlarge".format(angle))
IJ.run(ch2, "Rotate...", "angle={} interpolation=Bilinear enlarge".format(angle))
rm.reset()

# Prompt for bounding box
IJ.setTool("rectangle")
WaitForUserDialog("ROI Alignment",
    "Draw a rectangular bounding box on channel 1. Add it to ROI Manager, then click OK."
).show()

if rm.getCount() < 1 or rm.getRoi(0).getType() != Roi.RECTANGLE:
    IJ.log("Invalid bounding box. Skipping file: " + file_name)
```

```
        ch1.close()
        ch2.close()
        continue

    user_bbox_roi = rm.getRoi(0)
    transformed = transform_rois(pattern_rois, ref_bbox_roi, user_bbox_roi)

    # Save the newly transformed ROIs
    rm.reset()
    for r in transformed:
        rm.addRoi(r)

    roi_save_path = os.path.join(work_dir, "ROI", file_name.replace(".tif", "_ROIs.zip"))
    rm.runCommand("Save", roi_save_path)

    # Save processed channels
    FileSaver(ch1).saveAstiff(os.path.join(work_dir, "Channel1", file_name))
    FileSaver(ch2).saveAstiff(os.path.join(work_dir, "Channel2", file_name))

    # Merge & save
    merged = RGBStackMerge.mergeChannels([ch1, ch2], False)
    FileSaver(merged).saveAsTiff(os.path.join(work_dir, "Merged", file_name))

    # Cleanup
    ch1.close()
    ch2.close()
    merged.close()
    rm.reset()

IJ.showMessage("Processing completed successfully!")
```

Supplementary Macro 3: Condensation Quantification

```
# Fluorescence Analysis in Fiji using Jython (Python)
from ij import IJ
from ij.io import DirectoryChooser
from ij.plugin.frame import RoiManager
from ij.plugin import Duplicator
from ij.measure import Measurements
from java.io import FileWriter, BufferedWriter
import os

# --- Prompt for folders ---
imgDir = DirectoryChooser("Select folder containing .tiff files").getDirectory()
roiDir = DirectoryChooser("Select folder containing ROI ZIPs").getDirectory()
outputFile = os.path.join(imgDir, "Fluorescence.csv")
maskOutputDir = os.path.join(imgDir, "Thresholded_Masks")

if not os.path.exists(maskOutputDir):
    os.makedirs(maskOutputDir)

# --- Create CSV output ---
writer = BufferedWriter(FileWriter(outputFile))
writer.write("Image,ROI,Signal_Area_um2,Total_Area_um2,%_Signal_Coverage\n")

# --- Process each .tiff image ---
for fileName in os.listdir(imgDir):
    if not (fileName.lower().endswith(".tif") or fileName.lower().endswith(".tiff")):
        continue

    imgPath = os.path.join(imgDir, fileName)
    imp = IJ.openImage(imgPath)
    if imp is None:
```

```
    print("Could not open image:", imgPath)
    continue

baseName = os.path.splitext(fileName)[0]
roiZip = os.path.join(roiDir, baseName + "_ROIs.zip")
if not os.path.exists(roiZip):
    print("ROI ZIP not found:", roiZip)
    imp.close()
    continue

# --- Create thresholded binary mask ---
imp_thresh = Duplicator().run(imp)
IJ.run(imp_thresh, "8-bit", "")
IJ.setAutoThreshold(imp_thresh, "RenyiEntropy")
IJ.run(imp_thresh, "Make Binary", "")
IJ.run(imp_thresh, "Convert to Mask", "")  # Lock into 0/255

# --- Save thresholded image ---
thresholdOutputPath = os.path.join(maskOutputDir, "Thresh_" + baseName + ".tif")
IJ.saveAsTiff(imp_thresh, thresholdOutputPath)

# --- Load ROIs ---
rm = RoiManager.getRoiManager()
rm.reset()
rm.runCommand("Open", roiZip)
rois = rm.getRoisAsArray()
if not rois or len(rois) == 0:
    print("No ROIs found in:", roiZip)
    imp_thresh.close()
    imp.close()
    continue
```

```
    # --- Calibration for µm² ---
    cal = imp.getCalibration()
    pixelArea = cal.pixelWidth * cal.pixelHeight

    for roi in rois:
        roiName = roi.getName()

        # Total ROI area from original image
        imp.setRoi(roi)
        totalStats = imp.getStatistics(Measurements.AREA)
        totalArea = totalStats.area * pixelArea

        # Signal area (white pixels = 255 in mask)
        imp_thresh.setRoi(roi)
        stats = imp_thresh.getStatistics()
        hist = stats.getHistogram()
        signalPixels = hist[255]
        signalArea = signalPixels * pixelArea

        # % signal coverage
        percentCoverage = (signalArea / totalArea) * 100 if totalArea > 0 else 0

        row = "{},{},{:.3f},{:.3f},{:.2f}\n".format(fileName, roiName, signalArea, totalArea,
percentCoverage)
        writer.write(row)

    rm.reset()
    imp_thresh.close()
    imp.close()
```

```
writer.close()

# --- Post-step: Invert all saved masks (overwrite in place) ---
for fileName in os.listdir(maskOutputDir):
    if not fileName.lower().endswith(".tif"):
        continue

    filePath = os.path.join(maskOutputDir, fileName)
    imp_mask = IJ.openImage(filePath)
    if imp_mask is None:
        print("Could not open mask:", filePath)
        continue

    ip_mask = imp_mask.getProcessor()
    ip_mask.invert()
    imp_mask.updateAndDraw()

    IJ.saveAsTiff(imp_mask, filePath)
    imp_mask.close()

IJ.showMessage("Done", "Fluorescence.csv saved and all masks inverted in place.")
```

Supplementary Macro 4: HHD image analysis for hyphal, liquid and bacterial quantification

```
//Set line width to channel width
run("Set Measurements...", "area mean min perimeter redirect=None decimal=3");
run("Line Width...", "line=2800");
setTool("line");

//Get directory and Image name
//dir1 = getDirectory("Choose Source Directory ");
dir1 = getDirectory("image");
imageName = getTitle();
// Remove the file extension from the image name
dotIndex = indexOf(imageName, ".");
if (dotIndex != -1) {
   imageName = substring(imageName, 0, dotIndex);
}

//Duplicate image
run("Duplicate...", "duplicate");
rename("Image");

//Draw line
waitForUser("Draw line along the channel, then hit OK");
roiManager("Add");

//Split channels
run("Split Channels");

//Measure line green liquid
selectImage("C2-Image");
roiManager("Select", 0);
```

```
run("Plot Profile");

Plot.getValues(xValues, yValues);
csvFilePath = dir1 + imageName + "_liquid.csv";
file = File.open(csvFilePath);
print(file, "Distance_microns,Gray_Value");
for (i = 0; i < xValues.length; i++) {
    print(file, xValues[i] + "," + yValues[i]);
}
File.close(file);
//print("Plot profile data saved as: " + csvFilePath);
selectImage("Plot of C2-Image");
run("Close");

//Measure line red bacteria
selectImage("C3-Image");
roiManager("Select", 0);
run("Plot Profile");

Plot.getValues(xValues, yValues);
csvFilePath = dir1 + imageName + "_bacteria.csv";
file = File.open(csvFilePath);
print(file, "Distance_microns,Gray_Value");
for (i = 0; i < xValues.length; i++) {
    print(file, xValues[i] + "," + yValues[i]);
}
File.close(file);
//print("Plot profile data saved as: " + csvFilePath);
selectImage("Plot of C3-Image");
run("Close");
```

```
//Make hyphae into binary
selectImage("C1-Image");
run("Gaussian Blur...", "sigma=0.7 stack");
run("8-bit");
run("Sharpen");
run("Subtract Background...", "rolling=30");
waitForUser("adjust threshold", "test the threshold parameters in the window that next appears.");
run("Threshold...");
waitForUser("Click Apply then click OK");


run("Dilate");
run("Analyze Particles...", "size=100-Infinity pixel circularity=0.00-0.15 show=Masks");


//Measure line fungi
selectWindow("Mask of C1-Image");
roiManager("Select", 0);
run("Plot Profile");
Plot.getValues(xValues, yValues);
csvFilePathf = dir1 + imageName + "_fungi.csv";
filef = File.open(csvFilePathf);
print(filef, "Distance_microns,Gray_Value");
for (i = 0; i < xValues.length; i++) {
   print(filef, xValues[i] + "," + yValues[i]);
}
File.close(filef);
//waitForUser("Save plot data, then press ok");
selectImage("Plot of Mask of C1-Image");
run("Close");


//Measure red background
```

```
selectImage("C3-Image");
setTool("rectangle");
waitForUser("Draw Rectangle for background");
roiManager("Add");
roiManager("Select", 1);
roiManager("Measure");



//Measure green background
selectImage("C2-Image");
roiManager("Select", 1);
roiManager("Measure");

// Add a column called "fluo" and fill it with custom values
setResult("fluo", 0, "red");
setResult("fluo", 1, "green");
// Update the table
updateResults();

saveAs("Results", dir1+imageName+"_background.csv");

//Close everything
roiManager("Delete");
roiManager("Delete");
selectWindow("Results");
run("Close");
selectImage("Mask of C1-Image");
run("Close");
selectImage("C3-Image");
run("Close");
selectImage("C2-Image");
```

```
run("Close");
selectImage("C1-Image");
run("Close");
```

Supplementary Macro 5: Quantification of bacteria in liquid patches

```
//macro for liquid patches and bacteria


//rotate image

run("Line Width...", "line=3200");

setTool("line");


waitForUser("Draw line to identify the channel, then hit OK");


requires("1.33o");

getLine(x1, y1, x2, y2, width);

if (x1==-1)

exit("This macro requires a straight line selection");

angle = (180.0/PI)*atan2(y1-y2, x2-x1);

run("Arbitrarily...", "angle="+angle+" interpolate");


//Set measurements

run("Set Measurements...", "area mean min centroid perimeter shape redirect=None
decimal=3");


//Duplicate image

run("Duplicate...", "duplicate");

rename("Image");

run("Split Channels");

roiManager("Delete");


//aquire patch data


//Crop area of interest

selectImage("C1-Image");

setTool("rectangle");
```

```
waitForUser("Draw rectangle up to hyphal front, then hit OK");
roiManager("Add");
run("Crop");
selectImage("C2-Image");
roiManager("Select", 0);
run("Crop");
selectImage("C3-Image");
roiManager("Select", 0);
run("Crop");

//Make mask of green liquid
selectImage("C3-Image");
run("Convert to Mask");
roiManager("Delete");

//make ROI per liquid patch and measure area and coordinated
run("Analyze Particles...", "size=100-Infinity show=[Overlay Masks] display add composite");

//close green results
selectWindow("Results");
run("Close");

//measure bacteria pixel intensities
selectImage("C2-Image");
roiManager("Measure");

//save results
selectWindow("Results");
saveAs("Results", dir1+imageName+"_b_patch.csv");

//close everything
```

```
roiManager("Delete");
selectWindow("Results");
run("Close");
selectImage("C3-Image");
run("Close");
selectImage("C2-Image");
run("Close");
selectImage("C1-Image");
run("Close");
```
